# Alkali-metal alkoxides as additives in polar organometallic chemistry for M–H and M–X exchange reactions

**DOI:** 10.1039/d6cs00275g

**Published:** 2026-07-12

**Authors:** Neil R. Judge, Andreu Tortajada, Eva Hevia

**Affiliations:** a Departement für Chemie, Biochemie und Pharmazie, Universität Bern Freiestrasse 3 3012 Bern Switzerland eva.hevia@unibe.ch; b Département de Chimie, Université de Fribourg Chem. du Musée 9 1700 Fribourg Switzerland andreu.tortajadanavarro@unifr.ch

## Abstract

Typified by the Lochmann–Schlosser superbase, the use of Group 1 alkali-metal alkoxides as additives to enhance the reactivity of s-block organometallic reagents is a well-established concept in organic synthesis. However, for decades the origin of this activating effect remained unclear, and the constitution of the organometallic species involved was poorly understood. More recent studies have revealed that mixed-metal, mixed-aggregate complexes constitute the active species in these transformations. By enabling bimetallic cooperation, such combinations can display enhanced reactivity and distinct regioselectivities compared to their monometallic counterparts. This Tutorial Review highlights recent advances in this field, including the use of alkali-metal alkoxides in combination with organozinc and organomagnesium reagents to unlock new applications in deprotonative metalation and metal–halogen exchange reactions. Particular emphasis is placed on developing a mechanistic understanding of how these heterobimetallic mixtures operate. Focusing on recent synthetic studies on arene functionalisation, this Review also showcases the key role of coordination effects in finely tuning the regioselectivity of these transformations.

## Abstract 2

Alkali-metal alkoxides can transform main group reagents into cooperative heterobimetallic species, where aggregation and coordination strike the balance between reactivity and selectivity for useful synthetic transformation.

## Graphical abstract

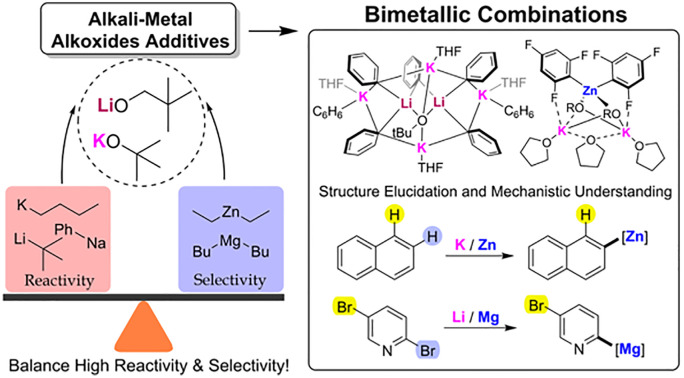

**Key learning points**

1. Understanding of how the addition of alkali metal alkoxides MOR (M = Li, Na, K) can have a dramatic effect on the reactivity of common organometallic reagents in synthesis such as organolithium, organomagnesium or main-group metal amides.

2. Rationalizing reactivity enhancement and special regioselectivities by the formation of heterobimetallic complexes.

3. Implications of alkali-metal and solvent effects on fine tuning structure/reactivity correlations.

4. Complex solution chemistry of the different heterobimetallic combinations containing MOR, which involve, in many cases, the presence of several organometallic species in equilibrium.

5. Synthetic potential and future applications in catalysis.

## Introduction

The use of alkali-metal alkoxides as additives to activate and boost the reactivity of s-block organometallics is an observation that was noticed for the first time over 70 years ago, where the addition of varying amounts of lithium or sodium alkoxides to organolithium or organosodium reagents was found to rapidly accelerate styrene and diene polymerisation.^1^ These findings rapidly evolved and led to the emergence of a wide range of powerful monometallic and bimetallic reagents combining alkoxides with organometallic reagents, which were later coined as ‘superbases’, because of their ability to carry out challenging deprotonative metalations (C–H to C–Metal). The more common combinations comprise an alkyllithium and a heavier alkali-metal alkoxide, typically sodium or potassium. The working hypothesis behind these superbasic reagents relies on the formation of mixed-metal/mixed-ligand aggregates, which boost their reactivity and stability compared to the monometallic reagents used to form the superbase. They have found a great applicability in synthetic chemistry, accessing new reactivity that is not possible by using just the monometallic components. To further improve their properties, this concept has been expanded to other less polar metals such as magnesium or zinc, whereby their pairing with an alkali-metal increased the reactivity and selectivity in the metalation of arenes and in metal-halogen exchange reactions to access synthetically relevant metalated species. This Tutorial Review aims to familiarise the readers with recent advances in this area of research, highlighting key advantages and existing limitations of these superbasic combinations and setting the challenges that these reagents are still facing. Special emphasis will be placed in shedding light on the constitution of these reagents, assessing how the nature of the alkali-metal and donor/solvent effects can finely tune their reactivities. Another goal of this review is to advance the fundamental understanding of the readers on bimetallic cooperation in s-block metal chemistry showcasing its enormous potential in organic synthesis and catalysis. Towards these objectives this Tutorial Review is divided into three main sections covering: (i) stoichiometric and catalytic applications of Group 1 metal heterobimetallic combinations; (ii) superbasic modifications combining alkali-metal alkoxides with organomagnesium and organozinc reagents to promote low polarity metalations: and (iii) extending alkoxide activation effects to metal/halogen exchange processes.

## Stoichiometric and catalytic applications of group 1 heterobimetallic combinations

### The development of the Lochmann–Schlosser superbase

**Fig. 1:**
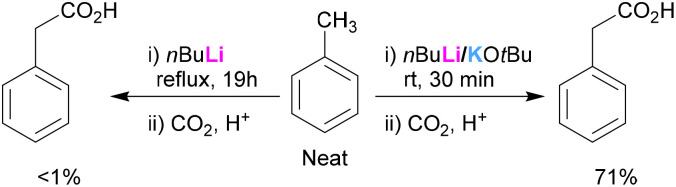
Enhanced reactivity of superbases in toluene metalation.

The archetypical example of ‘superbase’ type reagents is the famous Lochmann–Schlosser superbase, also known as ‘LiC-KOR’ base due to its *n*BuLi/KO*t*Bu composition. This combination of reagents was independently reported for the first time by Lochmann (1966)^2^ and Schlosser (1967),^3^ and since then they have become widely used basic combinations. A signature feature of this reagent is its unique reactivity in metalation reactions and their apparent simple composition, employing commercially available precursors which makes them widely accessible in any organic synthetic laboratory. Their enhanced metalating power can be exemplified in the benzylic deprotonation of toluene by *n*BuLi. In the absence of any additive, refluxing the organolithium reagent in toluene led to negligible conversion into the corresponding benzyl lithium,^4^ whereas in the presence of equimolar amounts of potassium *tert*-butoxide the metalation took place in less than 30 minutes at room temperature, delivering the corresponding carboxylic acid in good yield after quenching with CO_2_ (Fig. 1).^3^

**Fig. 2:**
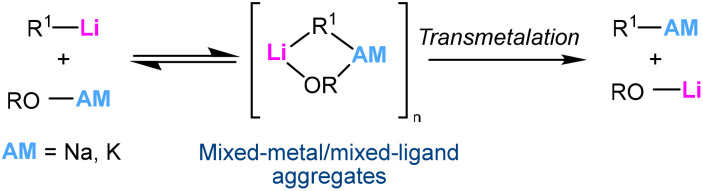
Mixed-metal/mixed-ligand aggregates.

In later studies, Schlosser states that although the combination of *n*BuLi and KO*t*Bu will eventually lead to the transmetalation of the two species (*n*BuK and LiO*t*Bu), driven by the high oxophilicity of Li and the formation of a highly insoluble alkylpotassium, it is unlikely that the highly reactive monometallic organopotassium species formed is the active base.^5^ This is due mainly to the fact that organopotassium reagents are known to be poorly soluble and decompose in ethereal solvents such as THF, even at –78 °C, whereas superbasic mixtures are known to be stable in these solvents up to –50 °C.^5^ Specially, alkyl potassium reagents are known to decompose *via* a β-hydride elimination, having made their preparation and characterization challenging. Furthermore, when the reactivity of organopotassium and LiC-KOR bases is compared, the latter provides generally higher yields with a greater control of the regioselectivity, hinting to a more complex constitution of these superbasic reagents. In fact, it is hypothesized that this unique reactivity and stability is due to the formation of mixed-metal/mixed-ligand aggregates, before the eventual transmetalation gives the new homometallic species (Fig. 2).

**Fig. 3:**
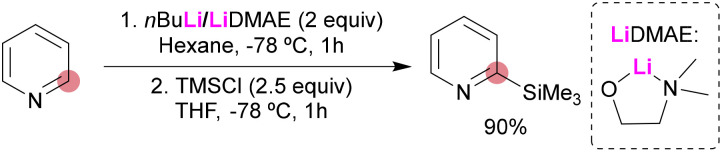
Metalation of pyridine by Caubère's base.

It is worth mentioning that the increased reactivity observed in these bimetallic systems is also observed when a lithium or sodium organometallic reagent is combined with an alkoxide with the same alkali-metal, again showing that the enhanced reactivity might be attributed to the formation of mixed-ligand aggregates. Caubère has described this combination as Unimetal Super Bases, where with the combinations of two reagents with the same alkali-metal a more reactive and soluble reagent can be obtained.^6^ Taking advantage of this, their properties could be exploited for the selective functionalisation of heterocycles, substrates that suffer from competitive addition of the organometallic reagents and are less robust upon metalation. For example, the metalation of pyridine in apolar solvents delivered the deprotonation in the 2-position in high yields as the only product (Fig. 3).^7^

**Fig. 4:**
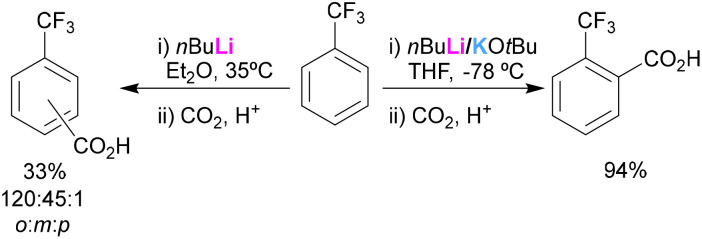
Regioselective metalation of trifluorotoluene by *n*BuLi/KO*t*Bu superbase.

Showcasing not only their higher reactivity but also their higher selectivity of superbases, Schlosser and coworkers reported that with a more activated substrate such as trifluorotoluene the outcome of the metalation reaction with the superbase and *n*BuLi was very different. The organolithium reagent was able in this case to metalate the aromatic compound in refluxing diethylether, but after carboxylation it showed a mixture of *ortho*, *meta* and *para* isomers with a total yield of 33%.^8^ However, when the superbase was used in THF at −78 °C, metalation occurs exclusively at the most acidic site of the substrate, furnishing exclusively the *ortho* substituted product with an excellent yield of 94% after carboxylation (Fig. 4).^9^

**Fig. 5:**
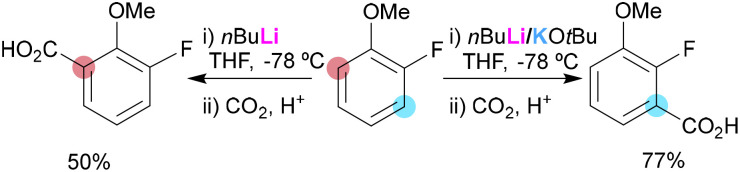
Regioselective metalation of 2-fluoroanisole.

Expanding the applicability of the superbase, Schlosser noticed a change in selectivity in the metalation of fluoroanisoles. Methoxy groups are a known directing group for ‘Directed *ortho* Metalation’ (D*o*M),^10^ where a Lewis donor group provides a coordinating site for the organolithium reagent, subsequently inducing its deaggregation and facilitating the metalation step *via* a complex induced proximity effect (CIPE).^11^ In the case of 2-fluoroanisole, the use of *n*BuLi at low temperatures allowed the selective metalation in the *ortho* position of the methoxy group in moderate yields, whereas the use of the superbasic mixture promoted the selective metalation in the *ortho* position to the fluorine substituent (Fig. 5), which is also the most acidic site in terms of p*K*_a_ in the fluoroarene.^8,9^ It is thought that the potassium alkoxide can provide a twofold activation of the organolithium reagent, forming the aforementioned mixed-metal/mixed-ligand ‘ate species whilst also enabling the deaggregation by acting as Lewis donors and form in solution smaller and kinetically more reactive species.^12^

### The LiNK base: an amide variant of the superbase

**Fig. 6:**
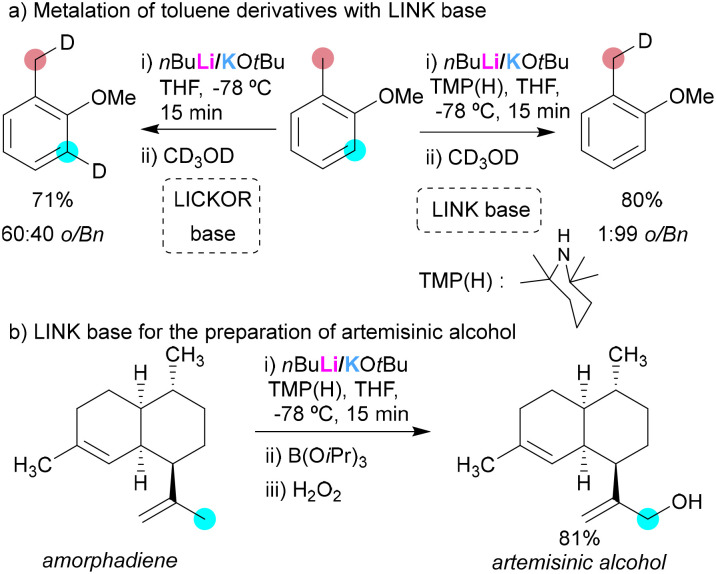
LiNK base for the regioselective metalation of C–H bonds. (a) Metalation of 2-methoxy toluene, (b) metalation of amorphadiene for the synthesis of artemisinic alcohol.

An important upgrade of the Lochmann–Schlosser superbase was developed more recently by O’Shea and coworkers by adding the amine TMP(H) (2,2′,6,6′-tetramethylpiperidine) to the LiCKOR base, making it a three-component mixture of *n*BuLi/KO*t*Bu/TMP(H), which has been coined as the LiNK reagent.^13^ Using this system, a profound difference in the regioselectivity in the metalations of substituted toluenes was observed. For example, in the metalation of 2-methoxytoluene with *n*BuLi/KO*t*Bu combination, a mixture of benzylic and *ortho* metalation is observed with poor selectivity, whereas with the LiNK base metalation is almost exclusively in the benzylic position (Fig. 6a). Previous reports from Schlosser have shown similar trends in the selectivity switch upon using lithium amides and potassium *tert*-butoxide combination.^14,15^ This change in selectivity has been rationalised by the ability of this base to induce an anion migration of the kinetic *ortho* metalated species to the benzylic site, as evidenced by NMR spectroscopic studies using substoichiometric amounts of TMP(H). More recently the LiNK base was shown to be compatible with more user-friendly conditions operating at 0 °C and room temperature in hydrocarbon solvents for the selective lateral metalations of toluenes, xylenes, and benzylsilanes.^16^ Showing the potential in synthetic chemistry, the use of this base was successfully used in the regioselective deprotonative borylation of allylic C–H bonds, achieving the synthesis of artemisinic alcohol (a precursor of the antimalarial artemisinin) in a multigram scale (Fig. 6b).^17^

**Fig. 7:**
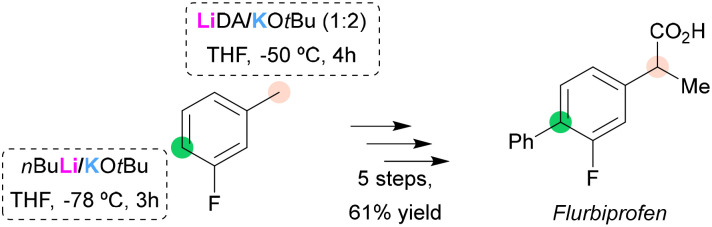
Regioselective metalation for the synthesis of flurbiprofen.

Showcasing the excellent synthetic potential of these bimetallic combinations, Schlosser has combined the different selectivity preferences of the superbases in a seminal paper for the preparation of the anti-inflammatory Flurbiprofen. Thus the sequential selective metalation of 3-fluorotoluene, firstly with the superbasic mixture *n*BuLi/KO*t*Bu followed by addition of LiDA/KO*t*Bu, furnished the synthesis of the desired drug in just 5 steps with an overall 61% yield (Fig. 7).^18^

### Superbases in organic synthesis: opportunities and limitations

**Fig. 8:**
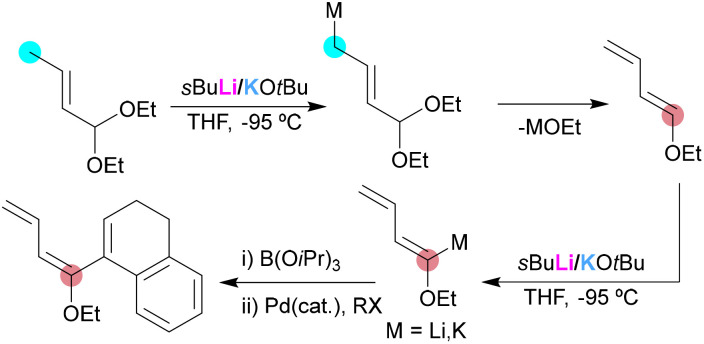
Regioselective metalation to form a metalated diene.

The use of superbases in organic synthesis has not been restricted to aromatic scaffolds, but it has been extended to other motifs. For example, Venturello reported the synthesis of metalated dienes from 1,1-diethoxybut-2-ene. A first metalation at cryogenic temperatures delivered the formation of the diene motive, which in the presence of an excess of superbase was metalated again to deliver the metalated diene (Fig. 8).^19,20^ This metalated intermediate was then used in the synthesis of tetrahydro-4*H*-pyran-4-ones and in palladium catalysed cross-coupling reactions to deliver substituted 1,3-dienes.

**Fig. 9:**
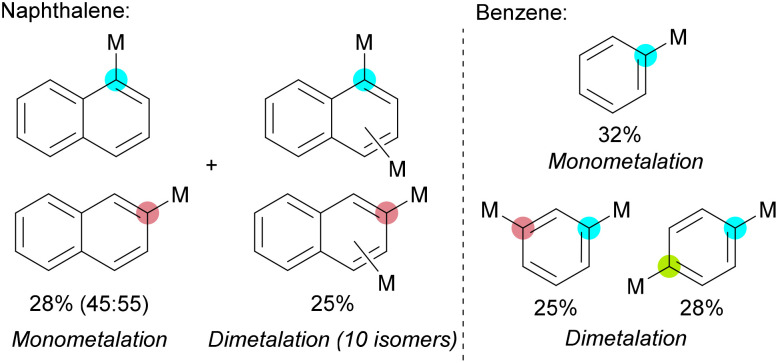
Unselective metalation with *n*BuLi/KO*t*Bu in hexane at −78 °C.

Despite their use in more efficient and selective synthetic protocols, superbases still face some limitations. For example, the metalation of non-activated substrates containing C–H bonds with very similar acidities results in the obtention of mixtures of different regioisomers or even the presence of multimetalated species as shown in Fig. 9 for the metalation of naphthalene and benzene.^5,21^ Moreover, the high reactivity of the metalated intermediates imposes some limitations on the functional group tolerance of these reagents, making them incompatible with the presence of more reactive groups such as carbonyl groups, nitriles or nitro groups. The use of ethereal solvents remains also restricted to low temperatures due to the decomposition of the base at higher temperatures, which might limit the use of this strategy at higher scale.

Overall, superbases have shown their synthetic potential in organic chemistry, demonstrating their higher metalating power than typical organolithium reagents, but with higher stability and solubility than the monometallic heavier alkali-metal organometallic reagents. However, most of the advances in the field have been empirical and the constitution of these reagents have been unveiled just in a scattered manner, hampering the development of these reagents to their full potential.

### Constitution of superbasic ROAM/RLi aggregates

**Fig. 10:**
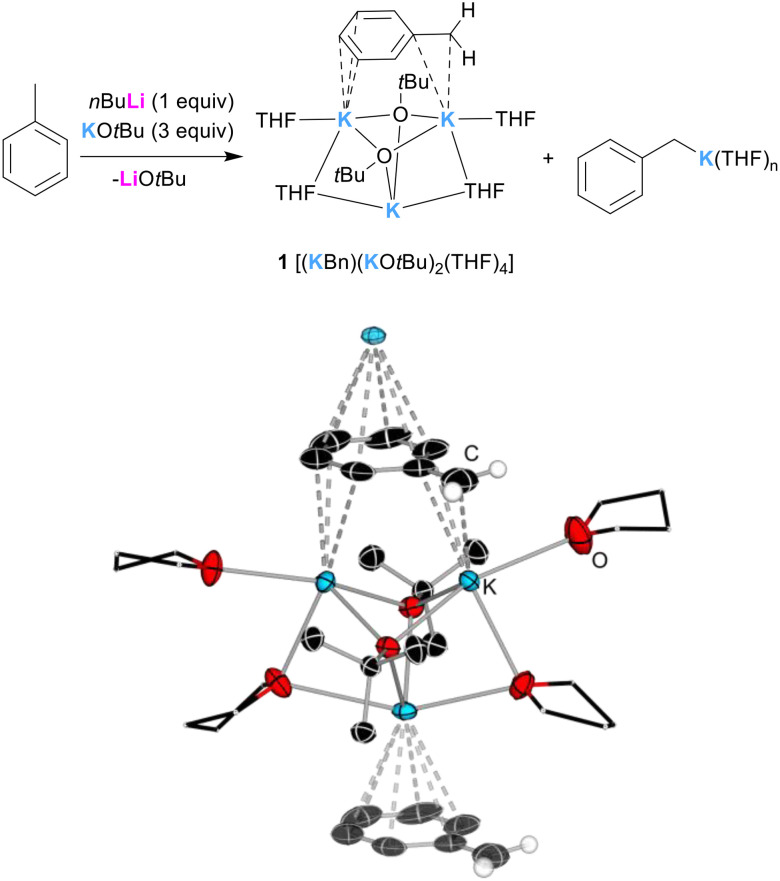
Toluene metalation resulting from LiCKOR superbase in the presence of excess KO*t*Bu and molecular structure of [(KBn)(KO*t*Bu)_2_(THF)_4_] (1).

Despite the general consensus regarding the synergic interaction of the mixed-metal/mixed-ligand aggregates that gives the highly reactive nature to these superbases, there is still a relatively limited understanding of the solution and solid-state behaviour of LiC-KOR superbase due to its poor solubility and high reactivity with ethereal solvents. If we think in monometallic species, moving from lithium to potassium the larger ionic radius and lower charge density of the metal lead to increasingly ionic and polarized M–C, M–N or M–O bonds, generally enhancing the nucleophilic and basic character. At the same time, significant changes in aggregation behaviour are observed, with lithium compounds often forming more covalent bonds with lower aggregation and higher solubility in hydrocarbon solvents, whereas sodium and potassium can form more ionic bonds and have higher aggregation and lower solubility. Regarding the bimetallic systems, Schlosser proposed that the special reactivity observed for these combinations could be due to the formation of multiple mixed aggregates of varying alkyl/alkoxide content in solution. One example of the multiple species that can form with these bimetallic reagents can be observed in the metalation of toluene by the superbase described earlier in this section. During the formation of benzyl potassium, employing 1 : 3 mixture of *n*BuLi and KO*t*Bu, Strohmann and coworkers have reported the crystallographic characterisation of a new aggregate [(KBn)(KO*t*Bu)_2_(THF)_4_] (1) resulting from the co-complexation of benzyl potassium with potassium *tert*-butoxide (Fig. 10).^22^ Lochmann reported the formation of similar adducts with excess potassium *tert*-pentoxide and benzyl potassium, giving after hydrolysis, toluene and the corresponding alcohol in a 1 : 2 ratio.^23^ They also reported more efficient metalation with higher concentrations of alkoxide or by using bulkier alkoxide than the *tert*-butoxide, again showing the different reactivity of the mixed-metal/mixed-ligand aggregates formed in solution.^24^

The first isolation of a well-defined monometallic alkoxide/alkyl aggregate was reported by Lochmann and Boche in 1990, where they described that the combination of *n*BuLi and LiO*t*Bu forms a tetrameric mixed aggregate [*n*BuLi·LiO*t*Bu]_4_ in the solid state.^25^ Albeit the homometallic nature of this structure, it offers some clues into the possible aggregates present in the mixed metal LiC-KOR system and this report laid the foundations for the subsequent spectroscopic and crystallographic investigations into these superbasic mixtures.

In 1993, Harder and Streitwieser reported the first mixed organosodium/lithium alkoxide aggregate characterized by X-Ray crystallography, forming a tetramer containing four lithium and four Na atoms, showing the preference of lithium to coordinate to the oxygen atom of the phenolate.^26^

**Fig. 11:**
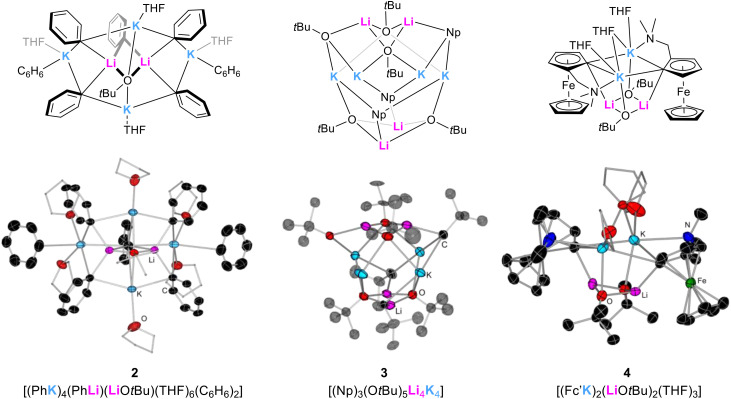
Structurally characterised Li/K mixed aggregate, giving structural insights into the composition of superbases. Molecular structures of [(PhK)_4_(PhLi)(LiO*t*Bu)(THF)_6_(C_6_H_6_)_2_] (2), [Li_4_K_4_(Np)_3_(O*t*Bu)_5_] (3) and [(Fc’K)_2_(LiO*t*Bu)_2_(THF)_3_] (4) are shown with displacement ellipsoids at 50% probability and all H atoms have been omitted for clarity.

Despite these early investigations, it was only in 2014 when one of the most relevant breakthroughs occurred, where a mixed-metal/mixed-ligand aggregate containing all components of a superbase was reported by Strohmann and coworkers.^27^ They were able to capture a snapshot into the metalation of benzene by the classical *n*BuLi/KO*t*Bu superbase in THF at –78 °C revealing a five component structure [(PhK)_4_(PhLi)(LiO*t*Bu)(THF)_6_(C_6_H_6_)_2_] (2) (Fig. 11, left). We can observe that lithium atoms form a 4-member {LiCLiO} central ring, showing the preference of the lithium atoms to form a harder interaction with the alkoxide fragment and a phenyl group, whereas the softer K atoms surround this ring forming π interactions in a perpendicular fashion to the *ipso*-carbon atoms of the metalated ring. The superbasic nature of this compound was confirmed by the formation of benzyl potassium at –40 °C in neat toluene. DFT calculations suggest that an initial coordination of the π system in toluene to the K atoms replaces the labile THF molecule and facilitates the deprotonation by bringing the substrate in close proximity to the anionic Ph moiety.

A fundamental aspect in the characterisation of superbase mixtures in solution is the relative low stability and solubility of these combinations in organic solvents often attributed to the formation of *n*BuK which is thermally unstable and can undergo β-hydride elimination. In 2016, Klett and coworkers sought to employ the more lipophilic neopentyl lithium (NpLi) reagent, which lacks of any β-hydrogen atoms, in combination with KO*t*Bu to hopefully reveal a more soluble and thermally stable superbasic mixture, analogous to the original Lochmann–Schlosser superbase with sp^3^-hybridized carbon anions. This combination afforded a white precipitate which was confirmed as NpK in a very moderate yield of 30%. Subsequent crystallisation experiments of the resulting mother liquor led to the characterisation of multiple mixed aggregates of the form [Li_4_K_4_(Np)_*n*_(O*t*Bu)_8−*n*_] (where *n* = 1, 2 or 3), where the authors note a significant decrease in the stability of the complex with increasing Np content.^28^ The first of the compounds to be characterised was the bimetallic [Li_4_K_4_(Np)_3_(O*t*Bu)_5_] (3) fragment (Fig. 11, middle). The structural make-up of this mixed aggregate has a remarkable resemblance to that of Mulvey's previously reported all alkoxide octameric [Li_4_K_4_(O*t*Bu)_8_] structure which adopts a breastplate arrangement.^29,30^ Remarkably, each Np group present in 3 is bonded to two K and one Li centres. Detailed spectroscopic studies by Klett in deuterated cyclohexane solutions, including DOSY NMR experiments,^28,31^ suggest the presence of multiple mixed-metal/mixed-ligand aggregates in solution with varying Np/O*t*Bu ratios. This seminal report by Klett further endorses Schlosser's initial considerations that several reactive bimetallic mixed aggregates can co-exist in solution within RLi/AM-OR superbase mixtures.^5^ Further exemplifying the role of the alkoxide, Klett and co-workers showed that by combining NpLi with potassium *tert*-amylate (KO*t*Am), a monometallic aggregate can be obtained, K_4_Np(O*t*Am)_3_, which allowed the tetra-metalation of ferrocene.^32^

More recently, in 2022, Strohmann has studied the constitution of a new superbasic mixture, obtained by the metalation of *N*,*N*-dimethylaminomethylferrocene by the LiC-KOR superbase,^22^ which afforded a heterobimetallic Li_2_K_2_ aggregate 4 containing ferrocenyl and *tert*-butoxide anions (Fig. 11, right). This molecular compound exhibits superbasic reactivity by deprotonating toluene or *N,N*-dimethylbenzylamine in the α-position. Remarkably, they could prepare an enantioenriched version of this base profiting from an enantioselective lithiation and co-complexation with potassium *tert*-butoxide, but the chirality could not be successfully transferred into other metalated products, highlighting the complexity of these systems.

Trying to get further information on the coordination and aggregation of organometallic species with alkali-metals, the use of superbasic combinations has allowed the preparation and characterization of a series of monometallic alkali-metal benzyl complexes of the heavier alkali metals (Na, K, Rb, Cs), showing the difference in aggregation and reactivity upon descending the group 1 of the periodic table.^33–36^

The aforementioned contributions have been integral to the current understanding of the modus operandi of the LIC-KOR superbase, yet their true constitution remains difficult to ascertain and is a topic of debate amongst the polar organometallic chemistry community.^12,31^ What we can be certain of is the diverse and structural complexity observed for mixed-metal/mixed-ligand superbase aggregates which can co-exist in equilibrium giving rise to the unique synergic reactivity observed for commonly used superbases.

### Catalytic application of superbasic reagents

**Fig. 12:**
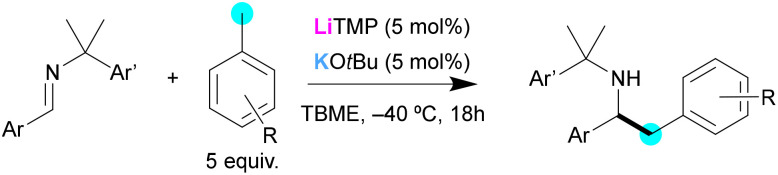
LiTMP/KO*t*Bu catalysed addition of toluenes to imines.

The superior reactivity and stability shown by superbases has allowed not only the advance in stoichiometric metalation of organic molecules, but it has also enabled the development of new catalytic reactions. Yamashita and Kobayashi have pioneered this field, showing that a combination of LiTMP with potassium *tert*-butoxide can promote the catalytic addition of toluene derivatives into imines, to form the corresponding substituted amines (Fig. 12).^37^ The catalytic conditions and the low temperature allowed the inclusions of fluoroarenes or pyridine groups in both the amine or the benzylic motive. This concept could be extended to allylic motives, using LiTMP/KO*t*Bu or LiTMP/NaO*t*Bu combination.^38^ Remarkably, when the amine motive was substituted with an alkene, the reaction performed better using the monometallic alkyl potassium reagents.^39^ The use of heavier alkali metal organometallic reagents in catalytic applications have flourished in recent years, due to the higher reactivity of these species when compared with the lighter organolithium reagents.^40^ Sodium, potassium or even caesium reagents have been used in catalytic reactions, such as hydrogen isotope exchange,^41–45^ alkene isomerisation,^46,47^ borylation^48^ or hydrogen transfer,^49^ showing the power of heavier alkali-metals.

## Superbasic modifications for low polarity metalation

Building on the first section of this Tutorial Review on Group 1 heterobimetallic reagents, this section showcases advances on exporting the concept of alkali-metal alkoxide activation to other polar organometallics, focussing on dialkyl and bis(amide) magnesium and zinc reagents.

### Structural studies on addition of alkali-metal alkoxides to organomagnesium and zinc reagents

**Fig. 13:**
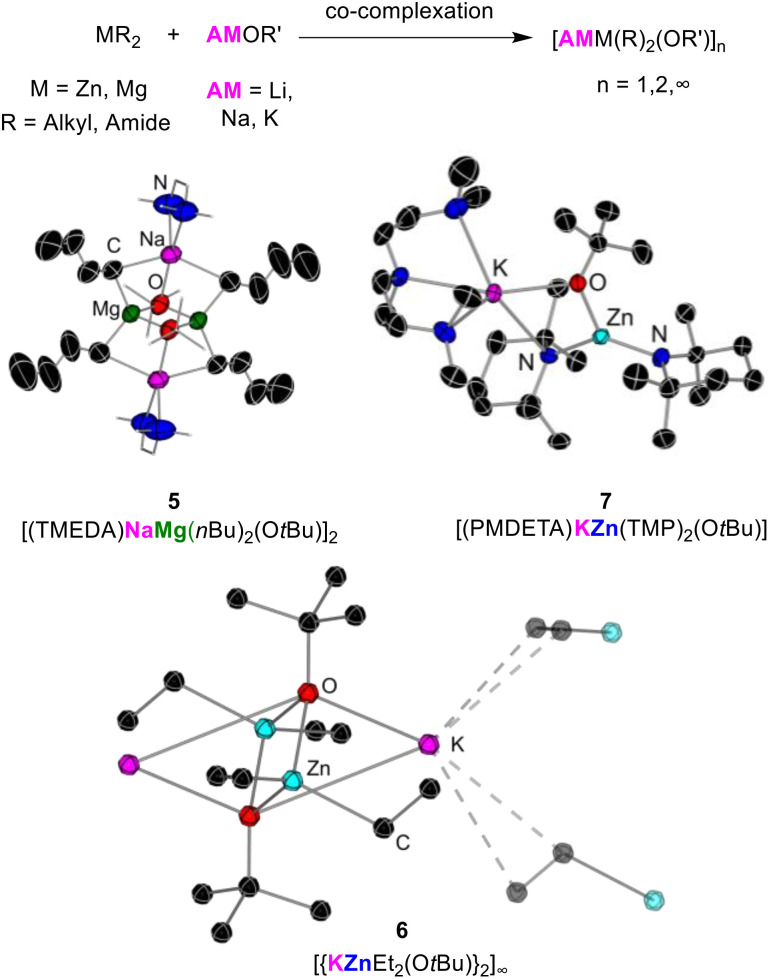
Co-complexation of alkali-metal alkoxides with diorgano-magnesium and zinc reagents.

Several structural studies have established the ability of alkali-metal alkoxides to form ‘ate complexes *via* co-complexation with bis(alkyl) and bis(amide) magnesium and zinc reagents. For example, early structural studies from Mulvey have shown that when NaO*t*Bu (or KO*t*Bu) is combined, or more accurately co-complexed, with *n*Bu_2_Mg in the presence of the Lewis donor TMEDA (TMEDA = *N,N,N*′*,N*′-tetramethylethylenediamine), formation of mixed alkyl/alkoxy sodium magnesiate [(TMEDA)NaMg(*n*Bu)_2_(O*t*Bu)]_2_ (5) is observed.^50^ Compound 5 (Fig. 13) exhibits a dimeric structure, featuring a central four-membered {MgOMgO} unit. Each Mg binds to two alkyl groups which are also bonded to one Na atom each. Interestingly each alkoxide bridges between the two Mg atoms and one Na atom, which achieves further coordinative stabilisation by binding to a chelating TMEDA ligand. Mulvey has also demonstrated that the formation of this type of bimetallic motif is not unique to the use of dialkyl magnesium reagents but can also be accessed with magnesium *bis*-diisopropylamide Mg(N(*i*Pr)_2_)_2_ forming [NaMg(N(*i*Pr)_2_)_2_(OR)]_2_ (R = *n*Oct, *n*Bu). However, in this case the mixed amido/alkoxy magnesiate is accessed through deprotonation of *n*-butanol or *n*-octanol with homoleptic sodium magnesiate, [NaMg(N(*i*Pr)_2_)_3_].^51^ It should be noted that no reactivity studies of these bimetallic systems towards organic molecules have been investigated.

Within organzinc chemistry, early studies from Richey have demonstrated that related mixed alkyl/alkoxy ate species can also be accessed by combining equimolar amounts of ZnEt_2_ and KO*t*Bu in toluene to furnish [{KZn(Et)_2_(O*t*Bu)}_2_]_∞_ (6).^52^ In this case the lack of a Lewis donor in the alkali-metal translates in the formation of a polymeric structure.

Illustrating the structural diversity of these bimetallic complexes, when using more sterically demanding anionic ligands monomeric motifs can also be obtained, as reported by Hevia for [(PMDETA)KZn(TMP)_2_(O*t*Bu)] (7) (Fig. 13) obtained by combination of Zn(TMP)_2_ and KO*t*Bu in the presence of tridentate donor PMDETA (*N,N,N*′*,N*″*,N*″-pentamethyldiethylenetriamine).^53,54^ In the case of 7, reactivity studies have confirmed that this alkali-metal exhibits superbasic behaviour (*vide infra*). While the constitution of the selected examples showcased in this section has been established by X-ray crystallographic analysis, it should be noted that NMR characterisation studies support that their bimetallic constitution is retained in solution.

### Alkali-metal alkoxide activation of organomagnesium species for deprotonative metalation

**Fig. 14:**
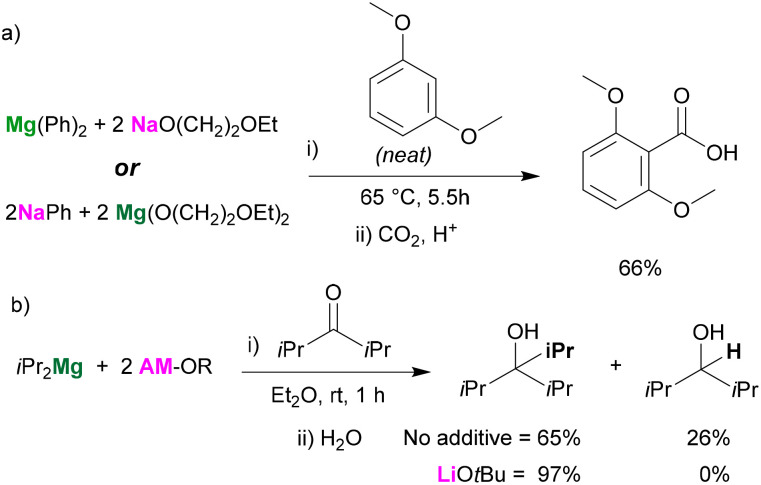
(a) Alkoxide promoted metalation of 1,3-dimethoxybenzene (b) alkali-metal alkoxide powered addition to ketones.

The popularity and advances of the LIC-KOR superbase have inspired the use of alkali-metal alkoxide as additives to organomagnesium and zinc reagents and the alkoxide containing bimetallic compounds discussed above can be conceptually described as modifications of the aforementioned Schlosser superbase. This type of reactivity was first described by Screttas in 1985, who employed more soluble alkali-metal 2-ethoxyethoxides (AMO(CH)_2_OEt) (AM = Li, Na, K) in combination with Ph_2_Mg (in a 2 : 1 ratio respectively). These bimetallic combinations can promote C2-metalation of 1,3-dimethoxybenzene (Fig. 14a) as well as the more challenging benzylic metalation of *m*-xylene and mesitylene, although elevated temperatures (70 °C) are required in order to achieve moderate to good yields (34–66%).^55^ Furthermore, it is also necessary the use of the substrate as the reaction solvent. Remarkably, in the absence of the sodium alkoxide the metalation process is completely inhibited. Similar yields are also achieved if an alternative bimetallic combination is employed by reacting PhAM (AM = Na, K) with magnesium alkoxide (Mg(O(CH_2_)_2_OEt)_2_) (Fig. 14a). While in this early study the authors did not isolate or characterise any putative organometallic intermediate, the formation of mixed-metal/mixed-ligand aggregates is proposed as a rational for the enhanced stability and solubility in hydrocarbon solvents of these bimetallic combinations.^55–57^ A related activating effect was also noted by Richey when assessing the use of alkali-metal alkoxide as additives to enhance the reactivity of *i*Pr_2_Mg towards the alkylation of ketones.^58^ In this case the alkali-metal alkoxides have an effect on the chemoselectivity of the reaction, favouring nucleophilic alkylation *versus* competitive formation of the relevant secondary alcohol due to a competing β-hydride elimination process (Fig. 14b). LiO*t*Bu and KOMe seemed to be the best partners for this C–C bond forming process, yet a clear and defined alkali-metal effect was not established in this case.

**Fig. 15:**
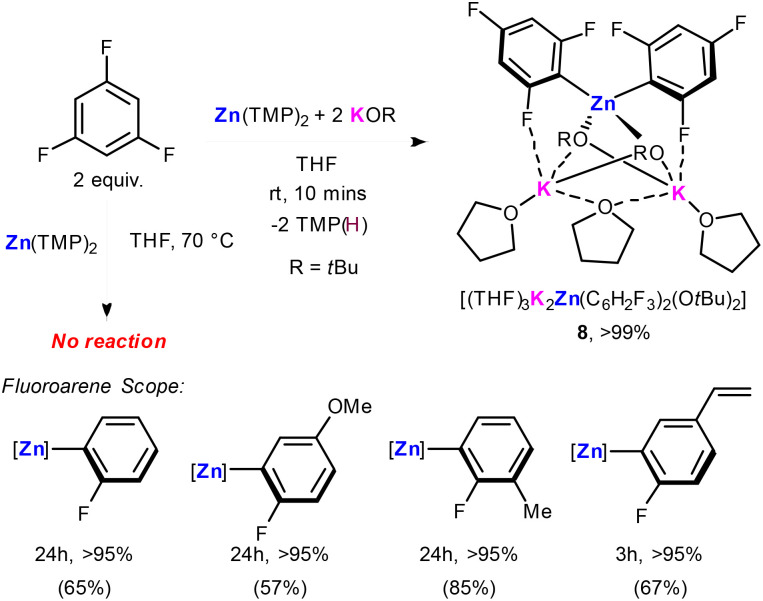
Zincation of fluoroarenes *via* Zn(TMP)_2_/2KO*t*Bu mixture to form higher order zincate (THF)_3_K_2_Zn(C_6_H_2_F_3_)_2_(OtBu)_2_ (8) and related complexes.

Within zinc chemistry, while previous studies have shown that zinc amides are versatile reagents to access zinc enolates by deprotonation of ketones and esters,^59–61^ they have shown no promise for the metalation of less activated aromatic substrates.^62^ Thus for example the amide Zn(TMP)_2_ fails to promote the zincation of 1,3,5-trifluorotoluene even under forcing reaction conditions (Fig. 15). However when two equivalents of KO*t*Bu are introduced this substrate can be selectively metalated at room temperature in THF to give potassium zincate [(THF)_3_K_2_Zn(C_6_H_2_F_3_)_2_(O*t*Bu)_2_] (8) in quantitative yields.^53^ Isolation and structural authentication of 8 demonstrated that this metalation is a genuine zincation, with both TMP groups being active towards the Zn–H exchange process. Furthermore, the potassium alkoxide has now been integrated into the constitution of the metalation product, with both O*t*Bu coordinating to the zinc centre. Remarkably each K atom binds to the two alkoxide ligands and interacts with the F atom which is located at the *ortho*-position to the C that has experienced the metalation. The authors propose that the distinct bonding preferences of this heterobimetallic system (Zn–C *vs.* K–F) must contribute to the overall stability of 8, which is stable at room temperature in THF solution for days without observing decomposition. This robustness contrasts with the thermal fragility of metalated fluoroarenes when using *n*BuLi or the Lochmann–Schlosser reagent as base, which require the use of cryogenic temperatures in order to avoid unwanted side reactions, such as benzyne formation and auto-metalation.^9,63^ These studies also uncovered an important alkali-metal effect, since reacting Zn(TMP)_2_ and 1,3,5-trifluorobenzene in the presence of two equivalents of LiO*t*Bu or NaO*t*Bu resulted in noticeable lower yields of metalation (42 and 69% respectively). The importance of the TMP group is exemplified by switching to a combination of Zn(HMDS)_2_ or ZnEt_2_ with KO*t*Bu which results in less effective metalation of 1,3,5-trifluorobenzene (46 and 43% respectively).

This approach was found to be versatile in terms of substrate scope allowing efficient zincation of a wide range of fluoroarenes including fluorobenzene, fluoroanisoles, fluorotoluenes and 4-fluorostyrene (see Fig. 15).^53^ Reactions took place with excellent regioselective control, favouring zincation of a C–H *ortho*-disposed to fluorine atom. In all cases metalation products [(THF)_3_K_2_Zn(Ar^F^)_2_(O*t*Bu)_2_] could be isolated as white solids in high yields. Demonstrating their synthetic utility, these intermediates can be intercepted with a range of electrophiles, including Pd catalysed Negishi cross-couplings or copper catalysed allylation reactions.

**Fig. 16:**
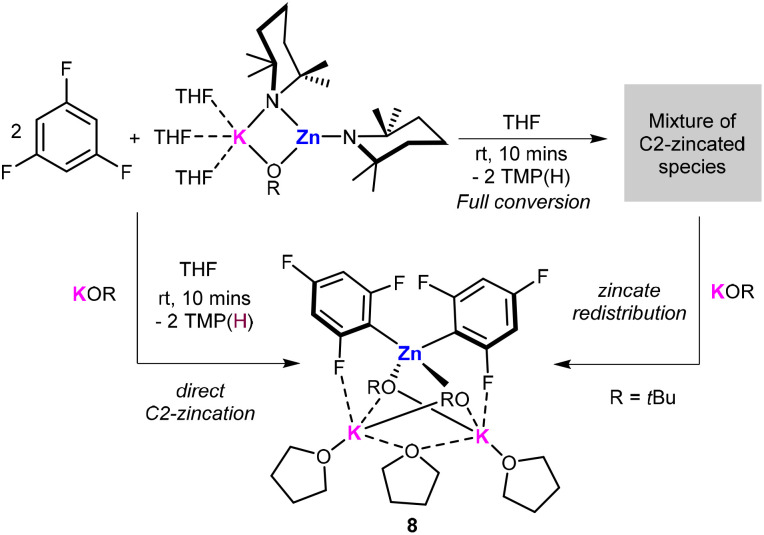
Role of second equivalent of KO*t*Bu in the metalation of two equivalents of 1,3,5-trifluorobenzene with lower-order potassium zincate (THF)_3_KZn(TMP)_2_O*t*Bu.

Investigations on the constitution of the bimetallic reagent performing these zincation reactions revealed a dual role for the potassium *tert*-butoxide. Of the two equivalents employed, only one undergoes co-complexation with the Zn amide to form the aforementioned potassium zincate [(THF)_3_KZn(TMP)_2_(O*t*Bu)] (Fig. 16) which could be isolated and structurally characterised as the PMDETA solvate 7 (see Fig. 13). This bimetallic complex is able to quantitatively metalate two equivalents of 1,3,5-trifluorobenzene, however NMR analysis of the reaction mixture prior to electrophilic interception showed the presence of a complex mixture of organometallic intermediates. Remarkably the second equivalent of KO*t*Bu affects the speciation of these species towards the exclusive formation of [(THF)_3_K_2_Zn(C_6_H_2_F_3_)_2_(O*t*Bu)_2_] (8) (Fig. 16). These findings add another layer of complexity to the use of the Group 1 metal alkoxides as additives in combination with organometallics. They suggest that the metal alkoxides may not only have an activating effect but also that they can instigate ligand redistribution processes, affecting the speciation of the organometallic intermediate(s) in solution.^12^ The latter effect can have important implications for further functionalisation of the metalated intermediates *via* electrophilic interception.

**Fig. 17:**
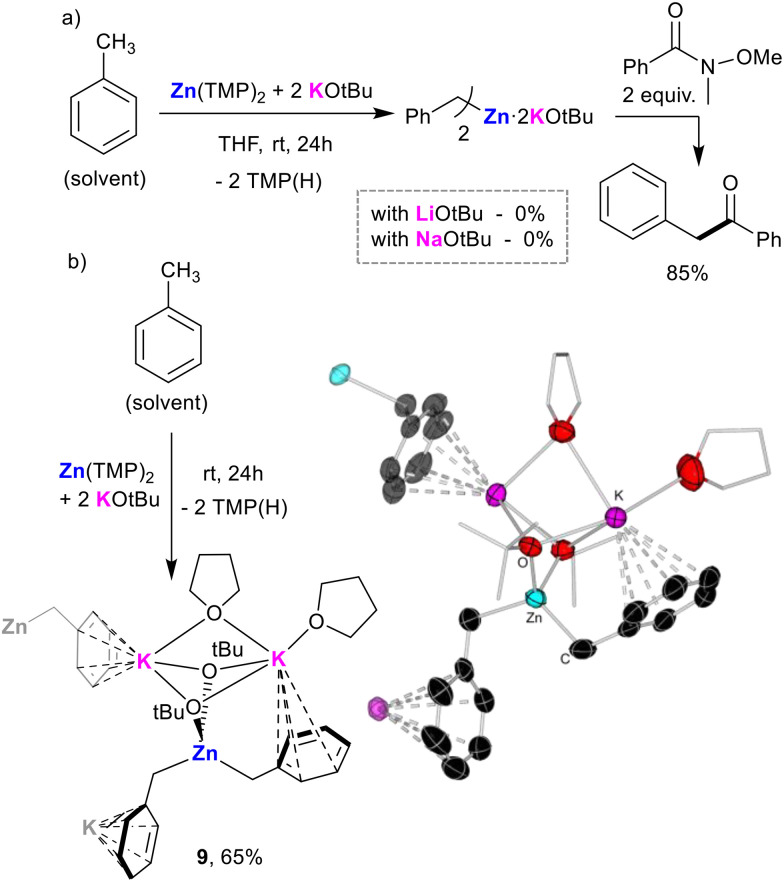
(a) Benzylic metalations of toluene and subsequent reaction of the metalated intermediates with Weinreb amides for benzylic aroylation (b) benzylic zincation of toluene using Zn(TMP)_2_/2KO*t*Bu.

This approach has also been extended to significantly less activated aromatic substates including benzene, naphthalene, toluene, xylenes and mesitylene.^53,64^ In some of these cases the substrate needs to be used as a solvent as shown in Fig. 17 for the lateral zincation of toluene forming [(THF)_2_K_2_Zn(CH_2_C_6_H_5_)_2_(O*t*Bu)_2_]_∞_ (9), where the benzylic unit can effectively be transferred to Weinreb amides to furnish the relevant 2-arylacetophenone (Fig. 17a).

The structure of 9 is related to that described for 8, comprising a mixed benzyl/alkoxide potassium zincate. A distinct bonding preference between the K and Zn centres with the benzyl anions is observed. Thus, while Zn forms two strong σ-bonds with the CH_2_ groups, each K centre prefers to π-engage with the aromatic ring of a benzyl group *via η*^6^ electrostatic interactions, giving rise to a polymeric structure. This reactivity is particularly remarkable when considering that Zn(TMP)_2_ is commercially sold as a 0.5 M solution in toluene. Surprisingly there is a pronounced alkali-metal effect when using the lighter alkali metal *tert*-butoxide congeners, LiO*t*Bu or NaO*t*Bu, completely shuts down the reactivity of the bimetallic mixture towards the non-activated alkylarenes, even at refluxing temperatures. This distinct reactivity has been attributed to the larger size and softer character of K *vs.* Li and Na, favouring the initial π-coordination between the zincate base and the aromatic substrate, which is proposed to be key in order to promote the Zn–H exchange process. In this regard, Pardue *et al.* probed the benzylic deprotonation of toluene by alkali metal amides *via* DFT calculations concluding that the aforementioned cation–π interactions facilitate the C–H bond scission finding that the heaviest alkali metal Cs amide offers the lowest energy barrier for this transformation.^65^

**Fig. 18:**
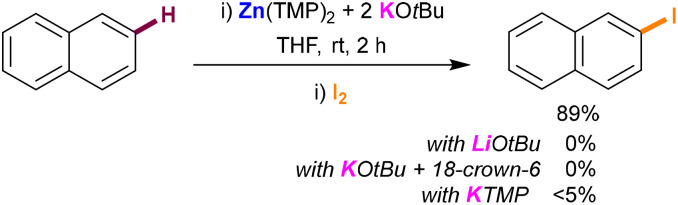
Contrasting naphthalene metalation between the LIC-KOR superbase and a Zn(TMP)_2_/2KO*t*Bu combination.

Showcasing the greater selectivity of this bimetallic combination in comparison with the Lochmann–Schlosser superbase (Fig. 9), when reacted towards naphthalene selective C2 zincation is observed, furnishing 2-iodonaphthalene in 89% yield after electrophilic interception with iodine.^64^ As previously noted by the toluene metalation, replacing KO*t*Bu by lithium or sodium *tert*-butoxide completely shuts down the reactivity. Furthermore, if the reaction is performed in the presence of two equivalents of the macrocyclic Lewis donor 18-crown-6, which can sequester the potassium atoms, the metalation is also suppressed. Collectively these results support that these reactions can be best described as potassium mediated zincations, where the alkali-metal is key for the success of the Zn–H exchange. Remarkably, it should be noted that KTMP is also not able to metalate naphthalene (Fig. 18).

**Fig. 19:**
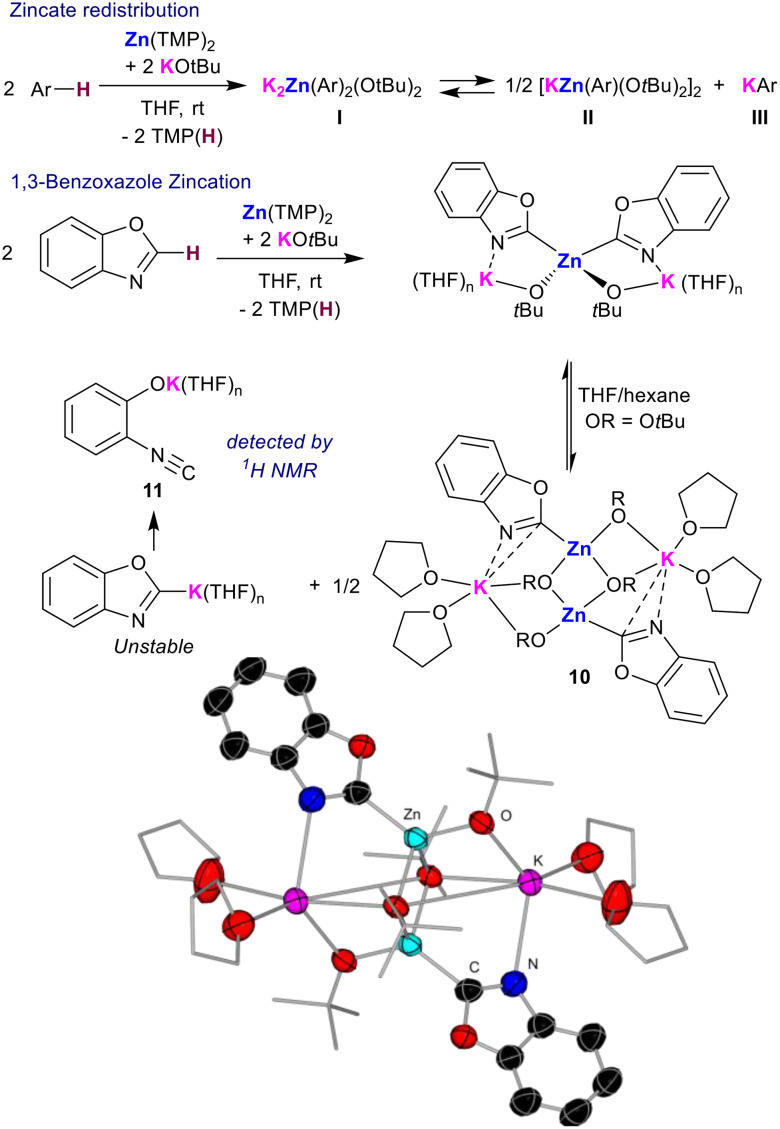
Zincate redistribution of higher order zincates [(THF)_*n*_K_2_Zn(Ar)_2_(O*t*Bu)_2_] (I) forming lower order zincates and potassium aryl species. Ar = C2-naphthyl, Ph and C2-benzoxazolyl.

This methodology could be extended to other notoriously challenging non-activated arenes under mild conditions enabling the regioselective C2-metalation of anthracene, C1-metalation of biphenylene and the monometalation of ferrocene. Unfortunately, the Zn(TMP)_2_/2KO*t*Bu combination was found to be incompatible with pyridine substrates and their derivatives resulting in substantial decomposition at room temperature. However, effective *alpha*-zincation of a range of more activated and sensitive five-membered heterocyclic molecules such as benzoxazole, 1-methyl-1,2,4-triazole and caffeine could also be achieved affording the relevant *alpha*-C-iodination products in excellent yields. Mechanistic studies on these systems have also revealed that the putative intermediates (THF)_*n*_K_2_Zn(Ar)_2_(O*t*Bu)_2_ (I) can be in equilibrium with the alkoxide rich potassium zincate [(THF)_*n*_KZn(Ar)(O*t*Bu)_2_]_2_ (II) and the relevant [(THF)_*n*_K(Ar)]_*n*_ (III) (Fig. 19, top). This equilibrium can be driven towards the formation of II over time due to the lack of solubility of III. This redistribution process has been mapped out for benzene and naphthalene, and it seems to occur at −30 °C when using hexane/THF solvent mixture. For 1,3-benzoxazole this zincate redistribution is observed at room temperature in d_8_-THF solutions although in this case the released potassium aryl species undergoes ring opening to form the corresponding C2-isocyanophenolate [K(1,2-O-C_6_H_4_-NC)] (11) along with [(THF)_2_KZn(C_2_-benzoxazolyl)(O*t*Bu)_2_]_2_ (10) which can be structurally characterised by X-ray crystallographic studies (Fig. 19).^66–69^ Formation of 11 is due to the limited stability of the relevant [K(C_2_-benzoxazolyl)] which rapidly undergoes ring opening. This decomposition path is well known in the literature for lithiated and magnesiated oxazoles.^66,67,69^

**Fig. 20:**
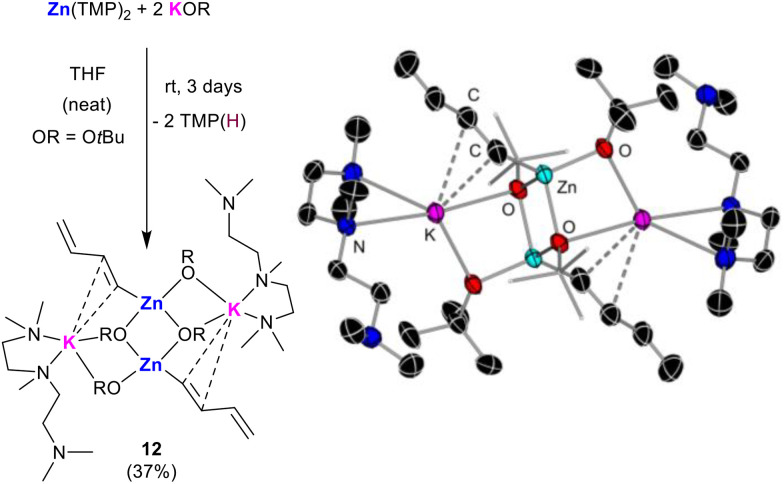
Decomposition of THF using Zn(TMP)_2_/2KO*t*Bu.

The high basicity of this bimetallic combination, made up of two seemingly weak bases, Zn(TMP)_2_ and KO*t*Bu, is also demonstrated by its ability to activate THF; the solvent employed in these reactions, affording the unique bimetallic product [(PMDETA)KZn(C_4_H_5_)(O*t*Bu)_2_]_2_ (12) (Fig. 20).^64^ The molecular structure of 12 features an *s-trans-1*,3-butadienyl (C_4_H_5_^−^) fragment coordinated to a Zn embedded within a potassium zincate framework. Reactivity studies using d_8_-THF as a solvent, support that this fragment is formed as the result of alpha-metalation, ring opening and oxygen-excision of this donor solvent.

**Fig. 21:**
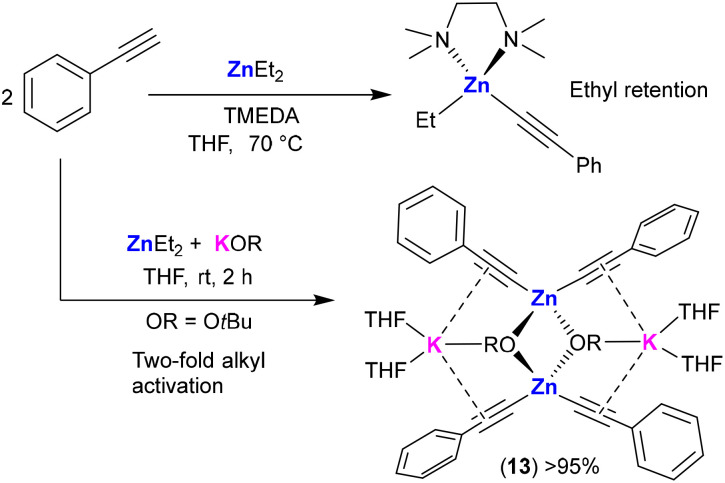
Activation of both ethyl groups in ZnEt_2_ using KO*t*Bu and subsequent reaction with phenylacetylene.

A related activating effect was also found when ZnEt_2_ was combined with equimolar amounts of KO*t*Bu.^54^ On their own dialkylzinc reagents are not regarded as particularly strong Brønsted bases due to their poor kinetic basicity. This has been largely attributed to the more covalent character of their Zn–C bonds when compared with other polar organometallics such as alkyllithium reagents.^70^ This can be illustrated by the reaction of ZnEt_2_ with two equivalents of phenylacetylene, in which only one of the Et groups is active towards deprotonation of the terminal alkyne, even under forcing reaction conditions, to furnish [(TMEDA)ZnEt(CCPh)] (Fig. 21). However, on the addition of one equivalent of KO*t*Bu to ZnEt_2_ in THF allows for the quantitative formation of [{(THF)_2_KZn(CCPh)_2_(OtBu)}_2_] (13) where both Et groups on Zn are active towards the Zn–H exchange process.

Collectively these studies evidence that, in a clear parallel with the Lochmann–Schlosser superbase, alkali-metal alkoxide additives can have profound activating effects on organomagnesium and zinc reagents to enhance their Brønsted basicity and increase their overall synthetic utility in the metalation/functionalisation of arene substrates. By forming alkali-metal ate complexes, these mixtures operate in a cooperative manner, offering reactivities and selectivities that cannot be replicated by their monometallic counterparts. Similarly, to Group 1 heterobimetallic combinations, the solution chemistry of these alkali-metal/magnesium (or zinc) mixtures, can be highly complex, with the presence of several mixed-metal/mixed-ligand organometallic species co-existing in solution.

## Extending alkoxide activation effects to metal–halogen exchange processes

### Alkoxide mediated activation of dialkyl magnesium and zinc reagents for metal/halogen exchange

Going beyond deprotonative metalation, alkali-metal alkoxides can also boost the reactivity of organomagnesium reagents towards metal/halogen exchange reactions.^12,71^ Pioneered by Gilman^72^ and Wittig,^73^ this transformation constitutes one of the most powerful methodologies to functionalise aromatic halides.^74^ Typically these reactions are mediated by polar organolithium reagents, although frequently they require the use of cryogenic conditions in order to control selectivity and improve functional group tolerance. Significant efforts have been devoted to the development of alternative methods employing less electropositive metals, such as Mg and Zn. Seminal advances in the field include Knochel's Turbo Grignard reagents (RMgX.LiCl) which can perform Mg/halogen exchange of an impressive range highly functionalised haloarenes under mild conditions with excellent chemoselectivity.^75^ Contrastingly, studies using dialkylmagnesium (R_2_Mg, R = alkyl) reagents have shown their limited potential to engage in Mg/halogen exchange reactions of arenes, except for substrates decorated with strongly electron-withdrawing groups.^76,77^

**Fig. 22:**
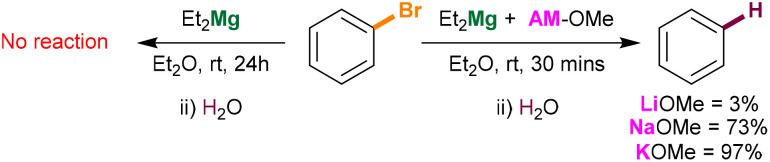
Alkoxide promoted Mg/Br exchange of bromobenzene using Et_2_Mg and alkali-metal methoxides.

Interesting, early studies from Screttas and Richey have shown that using stoichiometric amounts of alkali-metal alkoxides ramps up the reactivity of dialkylmagnesium reagents towards bromoarenes. Thus, Screttas reported that a combination of lithium 2-ethoxyethoxide (Li(O(CH)_2_OEt)) (two equivalents) and *n*Bu_2_Mg could successfully carry out the Mg/Br exchange of 4-bromoanisole at room temperature in a 50% yield.^78,79^ Richey reported a similar enhancement of the reactivity of Et_2_Mg towards bromobenzene using various alkoxide additives more commonly used in organic synthesis such as LiO*t*Bu, NaOMe, KOMe or KOPh (Fig. 22).^80^ Dialkylmagnesium reagents such as Et_2_Mg are typically inert towards such substrates, yet Richey realised an almost quantitative Mg/Br exchange of bromobenzene using a mixture of Et_2_Mg with the aforementioned AMOR additives. Interestingly, a pronounced alkali-metal effect was observed using the alkali-metal methoxides where KOMe performed the Mg/Br exchange in a quantitative fashion (>95%) compared with LiOMe which offered just 3% conversion of bromoarene under the same conditions (Fig. 22). The special reactivity of these mixtures was attributed to the formation of mixed alkyl/alkoxy alkali-metal magnesiates, however information on the constitution of these mixtures and the exact role of the alkali-metal remained elusive.

**Fig. 23:**
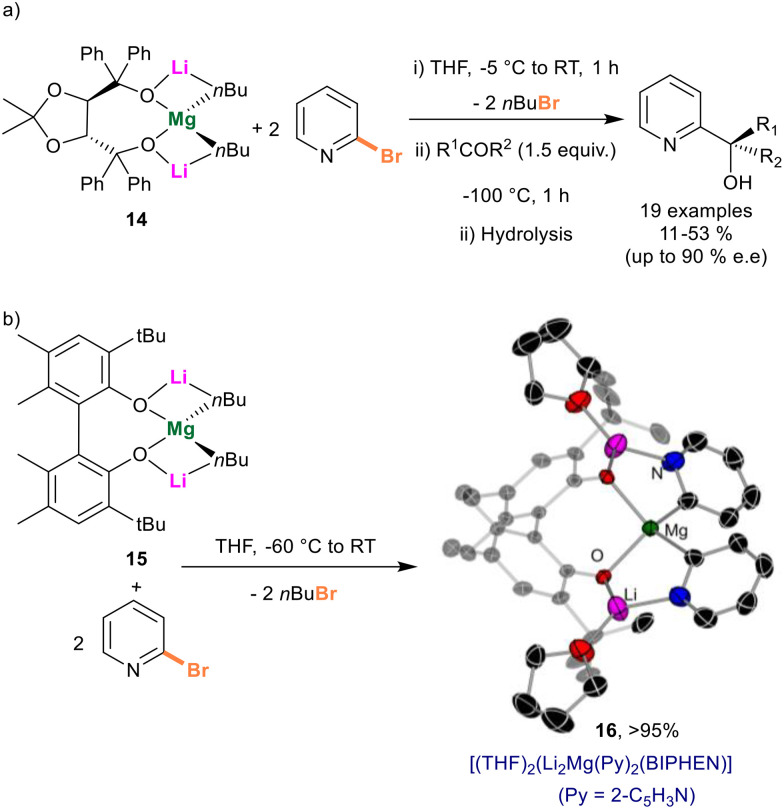
(a) Mg/Br exchange of bromopyridines by [Li_2_Mg*n*Bu_2_(R, R-TADDOL)] (14) (b) structurally mapping Mg/Br exchange of 2-bromopyridine with [Li_2_Mg*n*Bu_2_((*rac*)-BIPHEN)] (15) forming [(THF)_2_Li_2_Mg(Py)_2_(BIPHEN)] (Py = 2-pyridyl) (16).

More recently, Gros and Mongin have also introduced the use of chiral alkoxides to form mixed Li/Mg exchange reagents, generated *in situ* by deprotonation of chiral diol R,R-TADDOL (TADDOL = α,α,α,α-tetraphenyl-1,3-dioxolane-4,5-dimethanol) with higher order tetra(alkyl) Li_2_Mg*n*Bu_4_. This reagent allowed for the asymmetric synthesis of enantioenriched pyridyl carbinols (11–53%, up to 90% e.e) *via* Mg/Br exchange and subsequent addition to aldehydes (Fig. 23a).^81^ Compared with conventional organolithium reagents, these reactions can be carried out at room temperature in the presence of highly sensitive functional groups such as nitro substituents. A clearer insight into the constitution of the organometallic intermediates involved in these transformations has been provided by O’Hara using a racemic version of Li_2_-(*rac*)-BIPHEN (di)alkoxide in combination with *n*Bu_2_Mg forming lithium magnesiate [Li_2_Mg*n*Bu_2_((*rac*)-BIPHEN)] (15) (BIPHEN-H_2_ = 5,5′,6,6′-tetramethyl-3,3′-di-*tert*-butyl-1,1′-biphenyl-2,2′-diol). Treatment of this dilithium magnesiate with two equivalents of 2-bromopyridine afforded [(THF)_2_Li_2_Mg(Py)_2_(BIPHEN)] (16) (Fig. 23b) demonstrating that both *n*Bu were reactive towards Mg/Br exchange.^82^ Notably, in the molecular structure of 16, the pyridyl anions coordinate to the metals in an ambidentate fashion *via* a combination of Mg–C and Li–N bonds, contributing to the overall stability of this complex.

**Fig. 24:**
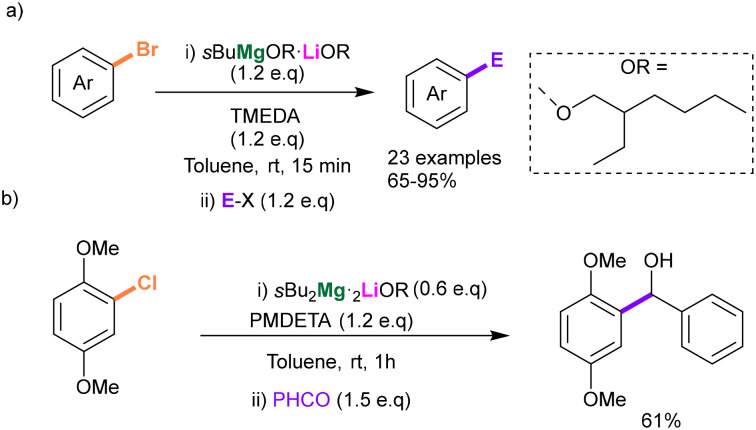
(a) Mg/Br exchange under ambient conditions in toluene promoted by soluble *s*BuMg(OR)·LiOR (OR = 2-ethylhexyl) (b) Mg/Cl exchange promoted by *s*Bu_2_Mg·2LiOR (R = 2-ethylhexyl).

Expanding further the synthetic potential of these approaches, Knochel has shown the power of combining *s*BuMg(OR) and LiOR (OR = 2-ethylhexyl) to generate *in situ* a wide range of aryl and hetero-aryl magnesiates *via* room temperature Mg/Br exchange (Fig. 24a). The use of a non-coordinating solvent such as toluene seems key for the success of this approach, hence a lipophilic long hydrocarbon chain alkoxide, derived from 2-ethylhexanol, needs to be employed to enhance solubility in this solvent. The subsequent magnesiated intermediates were found to react efficiently with a broad range of electrophiles such as aldehydes, ketones and allyl bromides whilst also participating in Kumada and Negishi (*via* transmetalation with ZnCl_2_) Pd-catalysed cross-couplings with aryl bromides/chlorides (Fig. 24a).^83^ An even more powerful exchange reagent containing two reactive alkyl ligands could be accessed combining 2 equivalents of LiOR with *s*Bu_2_Mg, upgrading these systems to perform the notoriously challenging Mg/Cl exchanges in the presence of Lewis donor PMDETA (Fig. 24b). For example, treatment of 2-chloro-1,4-dimethoxybenzene with *s*Bu_2_Mg·2LiOR (0.6 equiv.) at room temperature in toluene afforded (2,5-dimethoxyphenyl)(phenyl)methanol in a 61% yield after a quench with benzaldehyde (Fig. 24b) which is consistent with the activation of both *s*Bu groups towards Mg/X exchange.

**Fig. 25:**
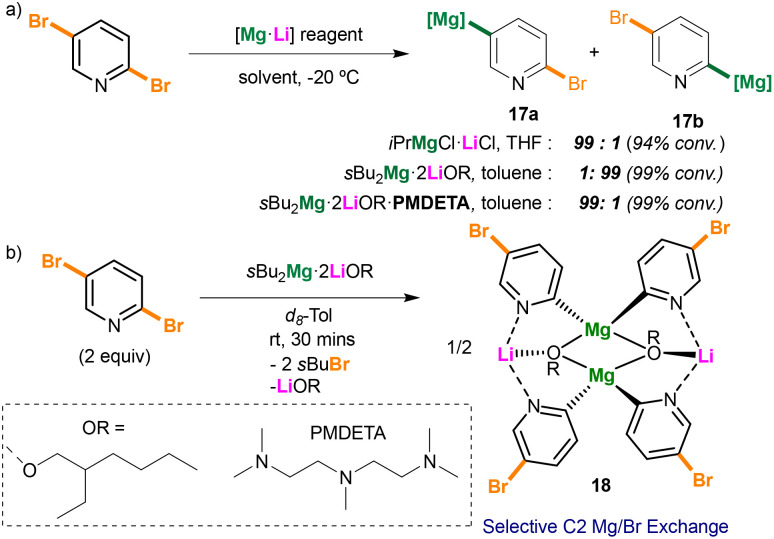
(a) Controlling C2- *versus* C5-selective Mg/Br exchange of 2,5-dibromopyridine in the presence of PMDETA (b) proposed mixed Li/Mg intermediate of C2 Mg/Br exchange.

Studies investigating the regioselectivity of these reactions using polyhalogenated substrates have uncovered the pivotal roles of coordination effects and bimetallic cooperation.^84^ This is exemplified for the reaction of 2,5-dibromopyridine with *s*Bu_2_Mg·2LiOR (R = 2-ethylhexyl) in toluene (Fig. 25a). If the tridendentate amine PMDETA is employed, regioselective Mg–Br exchange occurs at the C5 position, which is the most thermodynamically favoured position. The same regioselectivity is observed when the Turbo-Grignard reagent *i*PrMgCl·LiCl is used in the donor solvent THF. However, carrying out the reaction in the absence of PMDETA triggers a regioselectivity switch to exclusively promote the Mg/Br exchange at the C2 position. ^1^H NMR reaction monitoring studies support the formation of the intermediate [LiMg(OR)(2-C_5_H_3_NBr)_2_]_2_ (18) (Fig. 25b), where the Li atoms are stabilised by coordination to the pyridyl N, in a similar way as described above for 16. Based on these studies, it is proposed that the change in regioselectivity driven by the coordination preference of Li to the pyridine N, which can then guide the Br/Mg exchange to the C2 position. If a Lewis donor is added, this lithium-directing effect no longer operates and, as shown in Fig. 24, the selectivity of the Br/Mg exchange switches to the C5 position. This rationale can also explain why using the Turbo Grignard reagent in the ethereal solvent THF exclusively promotes the exchange at the C5 position.

**Fig. 26:**
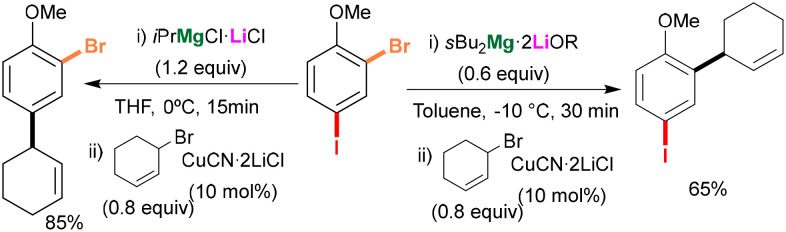
Coordination effects overriding the strength of C–X bond in selective Mg/Br exchange of 2-bromo-4-iodoanisole.

For this mixed-alkyl/alkoxide reagents, coordination effects can also be exploited to favour Mg–Br over Mg–I exchange. While *a priori*, considering only the degree of activation of the C–halogen bond, functionalisation *via* Mg–I exchange should be preferred.^85^ Treating 2-bromo-4-iodoanisole with *s*Bu_2_Mg·2LiOR in toluene, led to the unexpected regioselective Mg/Br exchange in the C2 position leaving the more reactive C–I untouched (Fig. 26). Indeed, using conventional Turbo Grignard reagents such as *i*PrMgCl·LiCl in THF exclusively leads to Mg/I exchange as expected.^84^ This special regioselective control is likely due to the absence of a donor solvent when using *s*Bu_2_Mg·2LiOR which allows the exchange reagent to interact with the methoxy substituent in the substrate, likely through dative Li⋯O interactions, favouring complex induced proximity effect (CIPE) type reactivity (see next section in this review).^11,86^

**Fig. 27:**
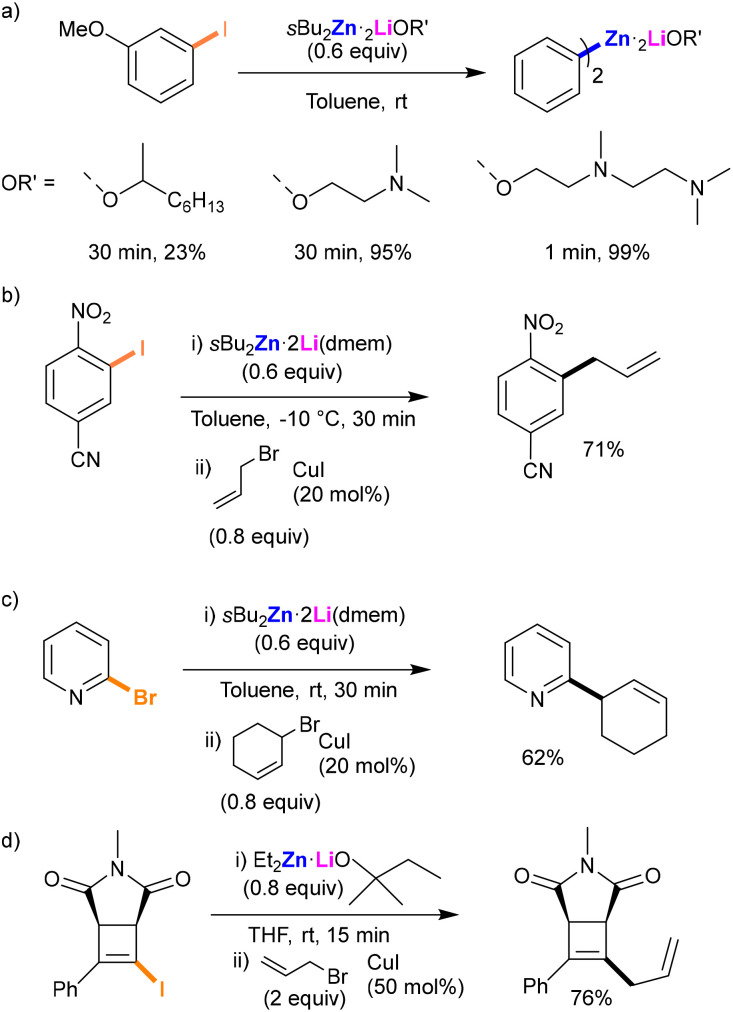
(a) Alkoxide effects in the Zn/I exchange of 3-iodoanisole using *s*Bu_2_Zn·2Li(dmem) (b) Zn/I exchange followed by Cu catalysed allylation of 3-iodo-4-nitrobenzonitrile (c) Zn/Br exchange of 2-bromopyridine at room temperature (d) Zn/I exchange using Et_2_Zn and lithium amylate combinations.

Contrasting with Li and Mg–halogen exchange, the use of Zn reagents in these transformations remains significantly underexplored.^87^ Compared with organolithium and Grignard reagents, less polar dialkylzinc (R_2_Zn) reagents are completely inert towards Zn/X exchange of aromatic halides.^87,88^ Several studies have shown that zincate modifications including lithium alkyl zincates R_3_ZnLi, and R_4_ZnLi_2_ can promote Zn/I exchange reactions towards aryl iodides although their reactivity towards aryl bromides is generally sluggish.^89,90^ Within this context Knochel has demonstrated that alkali-metal alkoxides can also boost the reactivity of dialkylzinc reagents towards Zn/X exchange with X = I, and Br.^88^ In these systems, a marked alkoxide effect was noted where the reactivity of the mixed alkyl/alkoxy zincate reagent, *s*Bu_2_Zn·2LiOR’, towards iodoarenes was significantly enhanced when N donor atoms were included in the alkoxide chain, with 2-{[2-(dimethylamino)ethyl]methylamino} ethoxide (dmem) containing two N-coordination sites delivering the quantitative Zn/I exchange of 3-iodoanisole in just one minute at room temperature (Fig. 27a). These reactions required the use of 2 equivalents of the alkoxide, Li(dmem), added to the particular dialkylzinc reagent, R_2_Zn (R = Et, *s*Bu, *t*Bu, *p*Tol), for the efficient Zn/halogen exchange to occur with a wide range of substrates. Due to the more covalent nature of the Zn–C bonds formed in these systems, an exceptional functional group tolerance was observed in which successful Zn/I exchange could be extended to highly functionalised iodoarenes decorated with sensitive NO_2_, CN and carbonyl moieties (Fig. 27b). Crucially, these bimetallic combinations could also promote the more challenging Zn/Br exchange for various bromoarenes, including thermally fragile bromopyridines (Fig. 27c). Demonstrating the synthetic utility of this system the polyfunctionalised mixed aryl/alkoxy zinc intermediates formed can be directly used in Pd catalysed Negishi cross-couplings and copper catalysed allylation reactions in excellent yields (Fig. 27b).^88^

Readily available Et_2_Zn can also be used in combination with lithium amylate to effectively perform the metal/halogen exchange of vinyl and (hetero)aryl iodides (Fig. 27d), forming the organozinc species at room temperature, which then can be intercepted with a range of electrophiles or cross coupling reactions to access a diverse library of functionalised building blocks.^91^

### Mechanistic studies of alkoxide mediated metal/halogen exchange reactions

In order to shed some light on how these bimetallic combinations operate and the key factors that are influencing the regioselectivity and enhanced basicity of these heterobimetallic mixtures, several studies have focused on the trapping and characterisation of the organometallic intermediates involved in these transformations prior to electrophilic interception. This section provides an overview on the progress made in this area.

**Fig. 28:**
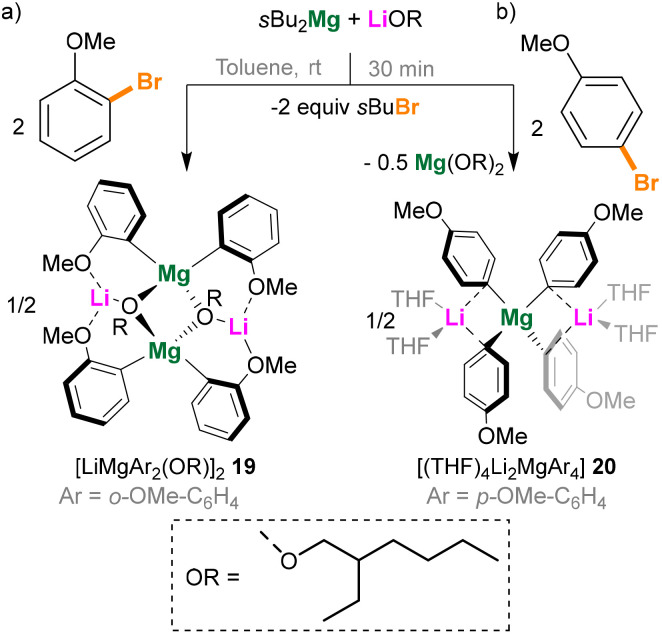
(a) Mg/Br exchange of 2-bromoanisole leading to [LiMgAr_2_(OR)]_2_ (Ar  = *o*-OMe-C_6_H_4_). (b) Mg/Br exchange of 4-bromoanisole leading to [(THF)_4_·Li_2_MgAr_4_] (Ar  = *p*-OMe-C_6_H_4_).

Using 2-bromoanisole as a model substrate, Knochel and Hevia have shown that the combination *s*Bu_2_Mg with two equivalents of the long chain alkoxide LiOR (R = 2-ethylhexyl) afforded the quantitative formation of dimeric mixed aryl/alkoxy lithium magnesiate [LiMgAr_2_(OR)]_2_ (Ar = *o*-OMe-C_6_H_4_) (19) (Fig. 28) with the concomitant formation of two equivalents of *s*BuBr.^92^ X-ray crystallographic analysis established the molecular structure of 19, where Mg is now coordinated to the *ortho*-C of two anisyl groups, occupying the position previously filled by a Br atom confirming the activation of both alkyl groups towards Mg/Br exchange. Interestingly, just one equivalent of LiOR was incorporated into the bimetallic constitution of the metalated intermediate 19. Indeed, identical reactivity towards 2-bromoanisole was observed using a 1 : 1 ratio of *s*Bu_2_Mg·LiOR indicating the second equivalent of the alkoxide was acting as a spectator in these reactions and the first equivalent was sufficient to enhance the reactivity of the dialkylmagnesium reagent. The molecular structure of 19 is completed with alkoxide bridges connecting the Mg and Li centres, however an important feature of the structure is the coordination between the methoxy groups of the metalated arene and the Li centres. This coordination environment helps to rationalise the regioselectivity previously discussed in Fig. 24 in which the absence of donor solvents leaves the Li atom in the exchange reagent available for *ortho* coordination offering selective C2 Mg/Br exchange in 2,5-dibromopyridine and *ortho* Mg/Br exchange in 2-bromo-4-iodoanisole leaving the more reactive C–I bond untouched.

Surprisingly, performing the same reaction with 4-bromoanisole, where the directing methoxy group is now remote to the Br substituent, resulted in the formation of a completely different metalated intermediate, namely the homoleptic tetra(aryl)lithium magnesiate [(THF)_4_·Li_2_MgAr_4_] (Ar = *p*-OMe-C_6_H_4_) (20) (Fig. 28). Compared to the mixed alkyl/alkoxy magnesiate 19, there is now no interaction between the Li centres and the methoxy groups in the substrate and 20 boasts a higher order Li : Mg ratio of 2 : 1. Crucially, there is also a distinct lack of alkoxide content in the constitution of 20, however NMR monitoring of this reaction confirmed that the quantitative Mg/Br exchange proceeds with concomitant formation of the corresponding Mg(OR)_2_ tying up the alkoxide ligand.

**Fig. 29:**
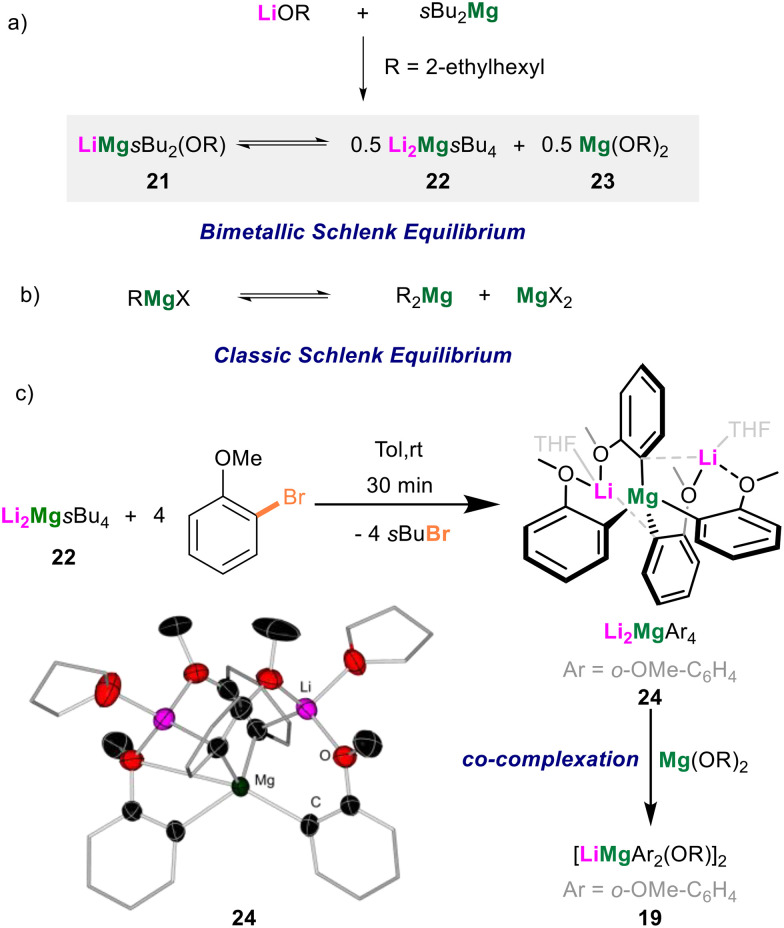
(a) Proposed equilibrium between [LiMg*s*Bu_2_(OR)] (21) with [Li_2_Mg*s*Bu_4_] (22) and Mg(OR)_2_ (23) (b) classical Schlenk equilibria (c) co-complexation between [Li_2_MgAr_4_] and Mg(OR)_2_ leading to [LiMgAr_2_(OR)]_2_ (Ar  = *o*-OMe-C_6_H_4_). (R = 2-ethylhexyl).

In order to rationalise the formation of these two distinct types of lithium magnesiates, a closer look was taken at the co-complexation between equimolar amounts of LiOR and *s*Bu_2_Mg. Detailed NMR spectroscopic studies revealed that indeed both components of the mixture self-assemble to form the expected mixed alkyl/alkoxy lower order lithium magnesiate LiMg*s*Bu_2_(OR) (21), however it is in equilibrium with Li_2_Mg*s*Bu_4_ (22) and Mg(OR)_2_ (23) (Fig. 29a). This equilibrium can be envisaged as a bimetallic version of the classical Schlenk equilibrium for Grignard reagents (Fig. 29b). The presence of this equilibrium provided a rationale for the formation of [(THF)_4_·Li_2_MgAr_4_] (Ar = *p*-OMe-C_6_H_4_) (20) resulting from the reaction of 4-bromoanisole with Li_2_Mg*s*Bu_4_, which, considering its all-alkyl constitution and higher-order composition (2 : 1 Li : Mg ratio), could be expected to be more reactive than LiMg*s*Bu_2_(OR) (21). In an effort to ascertain the active exchange species in this mixture, reaction of four equivalents of 2-bromoanisole with Li_2_Mg*s*Bu_4_ (prepared by co-complexation of a 2 : 1 mixture of *s*BuLi with *s*Bu_2_Mg) furnished [(THF)_2_Li_2_MgAr_4_] (Ar = *o*-OMe-C_6_H_4_) (24) (Fig. 29c) confirming its efficient and atom economical reactivity towards bromoarenes. Interestingly, this compound reacts with Mg(OR)_2_ (the other compound present in the proposed bimetallic Schlenk equilibrium depicted in Fig. 29a) to give mixed aryl/alkoxy lithium magnesiate [LiMgAr_2_(OR)]_2_ (Ar = *o*-OMe-C_6_H_4_) (19) which is the same species isolated from the reaction between 2-bromoanisole and the original exchange mixture of *s*Bu_2_Mg and LiOR (Fig. 27a). Crucially, the equilibrium depicted in Fig. 29a could be manipulated (by addition of excess Mg(OR)_2_) to exclusively form LiMg*s*Bu_2_(OR) (21), which was found to be unreactive towards 2-bromoanisole.

Thus, higher order tetraalkyl lithium magnesiate Li_2_Mg*s*Bu_4_ (22) is the most likely active species in the Mg/Br exchange process, facilitating the effective formation of Li_2_MgAr_4_ moieties, which, in the case of *ortho*-substituted aryl groups, undergo co-complexation to give [LiMgAr_2_(OR)]_2_ intermediates. Nevertheless, both types of organometallic intermediates 19 and 24 undergo further functionalisation by reacting with Weinreb amides or by transmetalation to ZnCl_2_ to undergo Pd catalysed cross couplings.^84,92^ Collectively, these mechanistic studies highlight the complexity of the constitution of the heterobimetallic species present in solution, which would remain concealed by merely carrying out *in situ* the organic electrophilic interception studies. Importantly, this work shows that using Knochel's *s*Bu_2_Mg·2LiOR mixture, the success of the Mg/Br exchange reactions can be explained by the presence of the more reactive bimetallic species Li_2_Mg*s*Bu_4_, rather than by the formation of a mixed alkyl/alkoxide lithium magnesiate, as initially thought.^83^

**Fig. 30:**
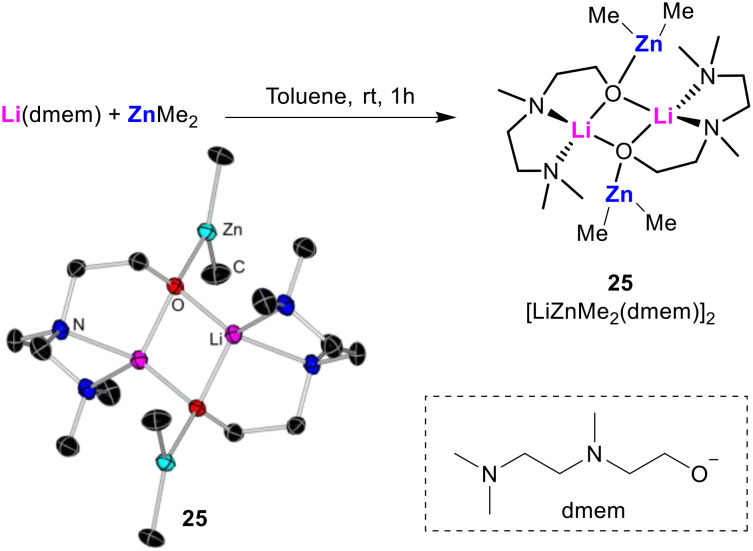
Co-complexation of Li(dmem) and ZnMe_2_ forming [LiZnMe_2_(dmem)]_2_ (25).

This type of bimetallic Schlenk equilibrium does not seem to be in operation for the combination of lithium alkoxides with dialkyl zinc reagents.^88^ Thus studies assessing the co-complexation of lithium 2-{[2-(dimethylamino)ethyl]methylamino} ethoxide (dmem), which contain two amido groups that can act as internal donors to the alkali-metal (see Fig. 30) in combination with ZnMe_2_ yielded dimeric mixed alkyl/alkoxy lithium zincate [LiZnMe_2_(dmem)]_2_ (25) which is stable in solution and does not undergo any ligand redistribution process.

**Fig. 31:**
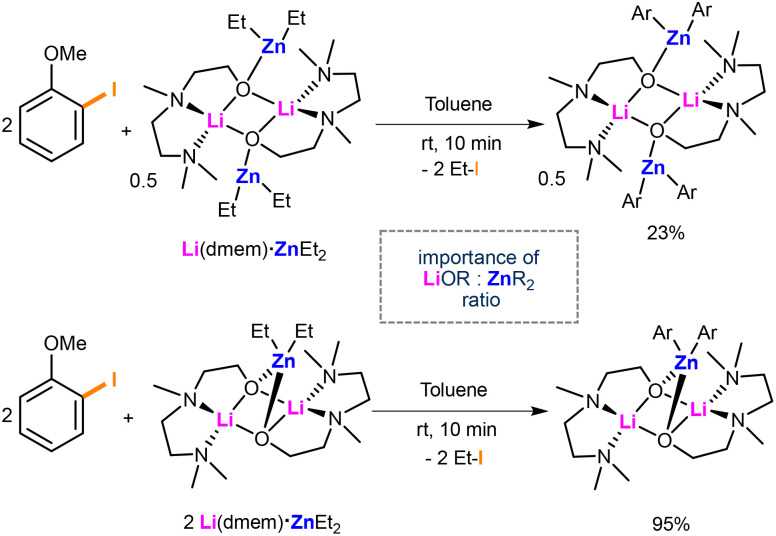
Importance of Li(dmem):ZnR_2_ (R = Ethyl) ratio for Zn/I exchange of 2-iodoanisole.

Likely this is due to the intramolecular stabilisation of the Li centres from the chelating N donor atoms in the alkoxide ligand (Fig. 31), forming a dimeric motif, where both Li centres are connected by the bridging O atoms of the alkoxide ligands, with each O binding also to a terminal ZnMe_2_ unit. While related bimetallic combinations have shown great promise for Mg/Br exchange processes (see Fig. 23), ^1^H NMR reaction monitoring studies with addition of variable amounts of Li(dmem) to a solution of Et_2_Zn and 2-iodoanisole in d_8_-toluene disclosed the importance of the LiOR/alkylzinc ratio for the success of the exchange (Fig. 31). While Et_2_Zn is completely inert towards exchange with 2-iodoanisole (2 equivalents), addition of 1 equivalent of Li(dmem) gave a conversion of only 23% after 10 min; on the other hand, with 2 equivalents of Li(dmem), the reaction is almost quantitative (95%). This is consistent with the formation of a more activated *bis*-alkyldialkoxyzincate species [Li_2_ZnEt_2_(dmem)_2_], where zinc is formally part of a more activated dianionic moiety. This differs drastically from the Li/Mg system discussed above where a hidden tetraalkyl lithium magnesiate species was responsible for the metal/halogen exchange. In this case, the alkoxide additive is a genuine feature of the active species carrying out the Zn/I exchange.

### Alkali-metal effects in magnesium/halogen exchange reactions

**Fig. 32:**
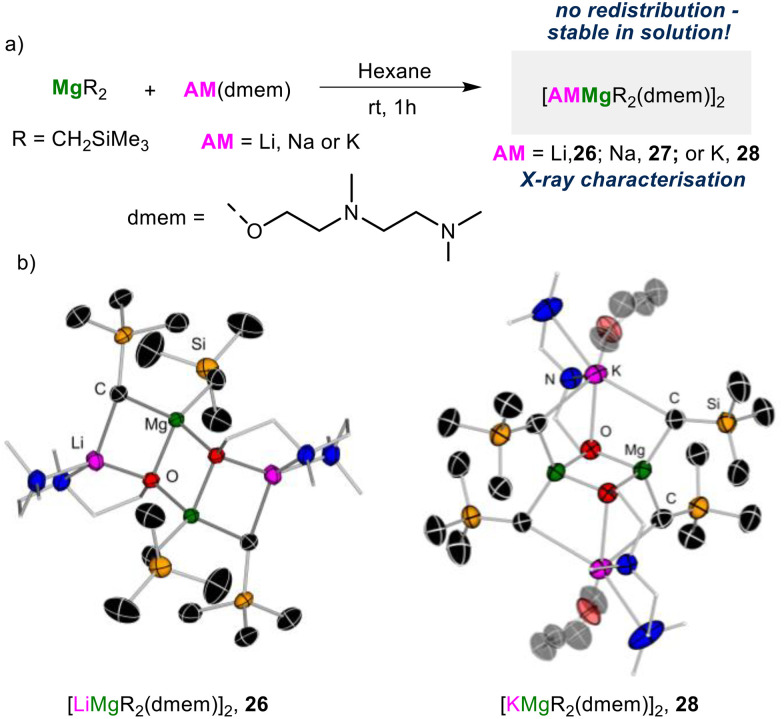
(a) Formation of mixed alkyl/alkoxy alkali-metal magnesiates [AMMg(CH_2_SiMe_3_)_2_(dmem)]_2_26–28 (b) contrasting molecular structures of lithium magnesiate 26 and potassium magnesiate 28.

Most of the examples showcased in this Tutorial Review are regarding Mg/X exchange reactions which combine a lithium alkoxide with a dialkyl(magnesium) reagent. The influence that the choice of alkali-metal can play in the stability and constitution of the organometallic intermediates, as well as on the ability of these systems to promote Mg/Br exchange reactions has also been investigated. Hevia has shown that complexation of equimolar amounts of AM(dmem) (AM = Li, Na, K) and Mg(CH_2_SiMe_3_)_2_ afforded a series of mixed alkyl/alkoxy alkali-metal magnesiates [AMMg(CH_2_SiMe_3_)_2_(dmem)]_2_ (AM = Li, 26; Na, 27; K, 28) (Fig. 32a). The structures of these compounds in the solid state have been established by X-ray crystallography whereas NMR spectroscopic analyses shows that these compounds are stable in C_6_D_6_ solutions and do not undergo redistribution processes as discussed above for the related mixed Li/Mg systems using the long chain LiOR (OR = 2-ethylhexyl).^93^Fig. 32b highlights their contrasting molecular structures upon altering the alkali-metal, where lithium magnesiate 26 boasts a ladder motif whereas the larger sodium and potassium congeners (27 and 28 respectively) form inverse crown like ring structures, comprising a cationic octagonal {(AMCMgC)}_2_^+^ ring which hosts two alkoxide anions in its core. Crucially, the coordination environment of Mg is identical in all three structures, however, the larger alkali-metals demand additional coordinative stabilisation and this gives rise to the ring structure where the electrostatic interactions between the alkali-metal and the alkyl groups is maximised, in the case of potassium magnesiate 28 an additional molecule of THF is required for stabilisation.

Interestingly, from these three alkali-metal magnesiates the K/Mg system 28 performed best towards Mg/Br exchange of model substrate 2-bromoanisole offering a 59% conversion in one hour at room temperature compared with 41% when the Li/Mg combination 26 is used. This could be increased to 75% using a 1 : 1 mixture of the potassium alkoxide K(dmem) and the more reactive dialkylmagnesium reagent, *n*Bu_2_Mg, highlighting the importance of the alkyl substituent used in these exchange reactions.

**Fig. 33:**
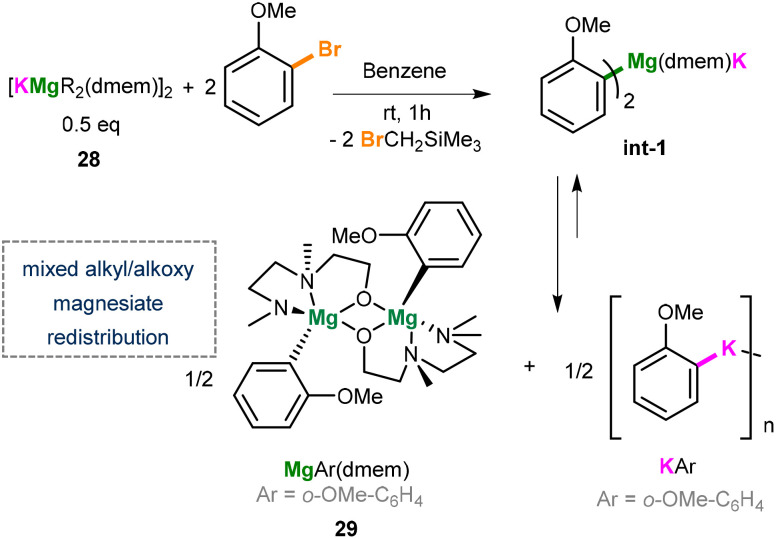
Redistribution of mixed aryl/alkoxy magnesiate int-1 into [ArMg(dmem)]_2_ (29) (Ar = *o*-OMe-C_6_H_4_) and [KAr]_∞_.

Adding another level of complexity to these reactions, when the reaction of [KMg(CH_2_SiMe_3_)_2_(dmem)]_2_ (28) with 2- bromoanisole was monitored by ^1^H-NMR spectroscopic studies, it was revealed that the product of Mg/Br exchange (KMg(Ar)_2_(dmem)) (int-1) (Ar = *o*-OMe-C_6_H_4_) (Fig. 33) undergoes dissociation into its homometallic counterparts mixed aryl/alkoxy magnesium species [ArMg(dmem)]_2_ (29) (Ar = *o*-OMe-C_6_H_4_) and potassium aryl species [KAr]_*n*_, which is highly insoluble in the employed reaction media. This ‘ate dissociation process may be driven by the poor solubility of the potassium species which is reminiscent to the equilibrium present in Lochman–Schlosser *n*BuLi/KO*t*Bu combinations, where an initial mixed metal alkyl/alkoxide aggregate is formed which eventually evolves into the exchange products LiO*t*Bu and *n*BuK.^5^ Ultimately, caution should be taken when using these bimetallic reagents in organic synthesis, since while 2-bromoanisole can be efficiently converted into anisole *via* Mg/Br exchange and aqueous quench, two entirely different organometallic species are formed which could be expected to have very different properties in terms of stability, reactivity and functional group tolerance, and could therefore affect further functionalisation of their aromatic moieties.

**Fig. 34:**
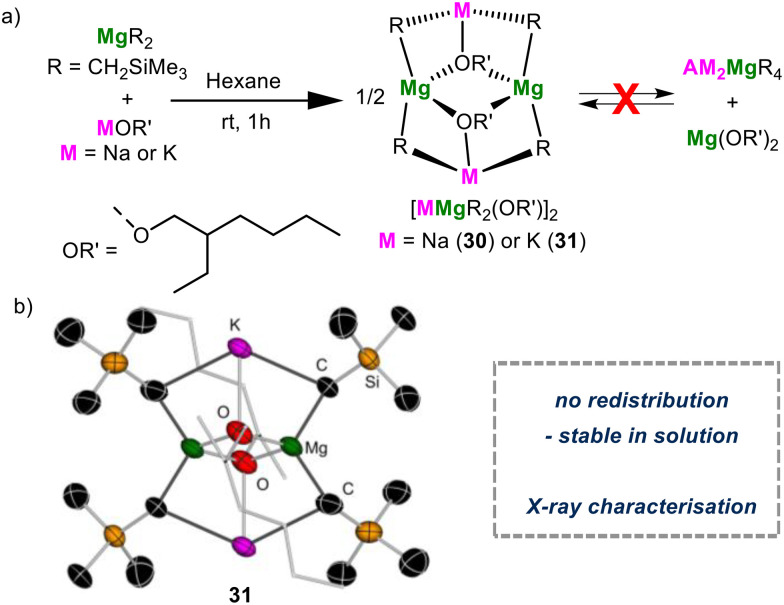
(a) Co-complexation of AMOR (AM = Na, K; OR′ = 2-ethylhexyl) with Mg(CH_2_SiMe_3_)_2_ and absence of bimetallic Schlenk equilibrium (b) molecular structure of potassium magnesiate [AMMg(CH_2_SiMe_3_)_2_(OR′)] (OR′ = 2-ethylhexyl) (31).

The importance of the nature of the alkali-metal was further underlined taking a step back and reevaluating the co-complexation of dialkylmagnesium reagents with Na and K long chain aliphatic alkoxide OR (R = 2-ethylhexyl). Thus, co-complexation of AMOR (AM = Na, K) with equimolar amounts of Mg(CH_2_SiMe_3_)_2_ led to the formation of mixed alkyl/alkoxy magnesiates [AMMg(CH_2_SiMe_3_)_2_(OR)]_2_ (AM = Na, 30; K, 31) (Fig. 34). These mixed alkyl/alkoxy magnesiates are perfectly stable in solution existing as single molecular entities without undergoing the bimetallic Schlenk equilibrium depicted in Fig. 29a, previously reported for [LiMg*s*Bu_2_(OR)]_2_ (R = 2-ethylhexyl).^92^ Remarkably, simply changing to the larger alkali-metal alkoxides, NaOR, and KOR completely shuts down the bimetallic Schlenk equilibrium process present with the smaller alkali-metal LiOR. Furthermore, reactions of mixed alkyl/alkoxy sodium and potassium magnesiates [AMMg(CH_2_SiMe_3_)_2_(OR)]_2_ (AM = Na, 30; K, 31) with 2 equivalents of 2-bromoanisole afforded the formation of mixed aryl/alkoxy metalated intermediates of the form [AMMg(Ar)_2_(OR)] (OR = 2-ethylhexyl). This is in stark contrast to the ‘ate dissociation process depicted in Fig. 33 above when using the chelating alkoxide and highlights how the choice of the alkoxide ligand in these reactions, along with the choice of alkali-metal can have profound consequences on the reactivity of these systems towards Mg/X exchange and the constitution of the metalated intermediates formed.

## Conclusions and outlook

Through a selection of recent examples from the literature, this Tutorial Review showcases the enormous synthetic potential of the use of alkali-metal alkoxides as effective additives to activate polar organometallic reagents. While separate pioneering studies in 1966 by Lochmann (with a focus on polymerisation) and Schlosser (with a focus on organic synthesis) established that the addition of equimolar amounts of KO*t*Bu to *n*BuLi can greatly boost the basicity of the alkyllithium reagents, it was almost 50 years later when an accurate tangible model for the constitution of this reagent as a mixed-metal mixed-alkyl-alkoxide cluster was postulated. Since then, the concept of alkali-metal alkoxide activation has been extended to other organometallic compounds such as organomagnesium, organozinc, and zinc amide reagents uncovering novel reactivity profiles with a wide range of applications in deprotonative metalation and metal/halogen exchange processes. Remarkably these systems can offer enhanced reactivities and unique regioselectivities which cannot be replicated by the single metal organometallic reagents. Mechanistic studies benefitting from the trapping of key organometallic intermediates and in-depth spectroscopic investigations have revealed that these special reactivities are underpinned by the formation of alkali-metal ‘ate complexes in which heterobimetallic cooperation must play a role. Adding further complexity, factors such as the choice of alkali metal, alkoxide ligand, solvent, and Lewis donor additives profoundly influence the speciation, reactivity, and regioselectivity of these heterobimetallic mixtures in solution.

As research in this area is likely to expand, these systems could deliver the next generation of transformative methodologies for selective synthesis and catalysis. This may include expanding the concept of alkali-metal alkoxide activation beyond the confines of main group chemistry, for example, by enhancing the kinetic reactivity of earth abundant transition metal alkyl and amide complexes towards deprotonative metalation or metal/halogen exchange processes, reactions typically considered the domain of highly polar reagents such as organolithiums. In this context, the redox flexibility of these transition metals, unavailable to lithium, may open up new opportunities for C–C bond formation. Furthermore, while the effects of using heavier alkali-metal alkoxides have often been noted in organic reactions, especially for caesium, there is still no consensus on their causes.

Another major challenge will be the translation of stoichiometric successes into general catalytic transformations. Although initial studies have demonstrated undoubted promise, the applicability of these bimetallic systems in catalysis remains largely confined to a narrow field of reactions. Widening this field would be particularly timely given the greater sustainability credentials of s-block metals over their precious transition metal competitors.

## Conflicts of interest

The authors have no conflicts to declare.

## Data Availability

No primary research results, software or code have been included and no new data were generated or analysed as part of this review.
